# Recent Progress in Nanomaterials for Modern Concrete Infrastructure: Advantages and Challenges

**DOI:** 10.3390/ma12213548

**Published:** 2019-10-29

**Authors:** Karla P. Bautista-Gutierrez, Agustín L. Herrera-May, Jesús M. Santamaría-López, Antonio Honorato-Moreno, Sergio A. Zamora-Castro

**Affiliations:** 1 Maestría en Ingeniería Aplicada, Facultad de Ingeniería de la Construcción y el Hábitat, Universidad Veracruzana, Calzada Ruiz Cortines 455, Boca del Río, Veracruz 94294, Mexico; karlapaty40@gmail.com (K.P.B.-G.); jsantamaria@uv.mx (J.M.S.-L.); antonio.honorato.ing@gmail.com (A.H.-M.); 2 Micro and Nanotechnology Research Center, Universidad Veracruzana, Calzada Ruiz Cortines 455, Boca del Río, Veracruz 94294, Mexico

**Keywords:** carbon nanotubes, cement-based materials, concrete infrastructure, graphene, graphene oxide, mechanical strength, nanomaterials, nano-Al_2_O_3_, nano-Fe_2_O_3_, nano-SiO_2_, nano-TiO_2_, smart infrastructure

## Abstract

Modern concrete infrastructure requires structural components with higher mechanical strength and greater durability. A solution is the addition of nanomaterials to cement-based materials, which can enhance their mechanical properties. Some such nanomaterials include nano-silica (nano-SiO_2_), nano-alumina (nano-Al_2_O_3_), nano-ferric oxide (nano-Fe_2_O_3_), nano-titanium oxide (nano-TiO_2_), carbon nanotubes (CNTs), graphene and graphene oxide. These nanomaterials can be added to cement with other reinforcement materials such as steel fibers, glass, rice hull powder and fly ash. Optimal dosages of these materials can improve the compressive, tensile and flexural strength of cement-based materials, as well as their water absorption and workability. The use of these nanomaterials can enhance the performance and life cycle of concrete infrastructures. This review presents recent researches about the main effects on performance of cement-based composites caused by the incorporation of nanomaterials. The nanomaterials could decrease the cement porosity, generating a denser interfacial transition zone. In addition, nanomaterials reinforced cement can allow the construction of high-strength concrete structures with greater durability, which will decrease the maintenance requirements or early replacement. Also, the incorporation of nano-TiO_2_ and CNTs in cementitious matrices can provide concrete structures with self-cleaning and self-sensing abilities. These advantages could help in the photocatalytic decomposition of pollutants and structural health monitoring of the concrete structures. The nanomaterials have a great potential for applications in smart infrastructure based on high-strength concrete structures.

## 1. Introduction

Construction engineering requires materials that enhance the mechanical properties of the cement-based composites for modern concrete infrastructure. For instance, the compressive, tensile and flexural strength of concrete structures need to be improved. For this, nanomaterials can be mixed with cementitious matrices to obtain concrete with high mechanical strength [1,2,3,4,5,6,7,8,9,10]. Nanotechnology can facilitate the development of nanomaterials incorporated into cement-based materials to increase their mechanical strength [11,12,13,14,15,16,17,18,19,20,21,22,23,24,25], decreasing their environment impact [26]. The CO_2_ emissions generated during the production of ordinary Portland cement can represent approximately between 5% and 7% of the world man-made emissions of this gas [27,28]. A main challenge of the cement industry is the reduction of the CO_2_ emissions. One alternative solution is the construction of concrete structures with higher mechanical strength and higher durability, which will decrease their maintenance requirements or need for early replacement. Thus, the concrete structures can have thinner sections, which will require less quantity of cement-based composites for their construction. 

The cement-based materials can be mixed with nanomaterials such as nano-silica (nano-SiO_2_), nano-alumina (nano-Al_2_O_3_), nano-ferric oxide (nano-Fe_2_O_3_), nano-titanium oxide (nano-TiO_2_), carbon nanotubes (CNTs), graphene and graphene oxide. In recent years, several researchers [29,30,31,32,33,34,35,36,37,38,39,40,41,42,43,44,45,46,47,48,49] have studied the incorporation of nanomaterials into cement-based materials. The mixture of cementitious composites and nanomaterials can increase the mechanical strength of the resulting concrete structures. Thus, the life cycle of these structures can be extended or they can require smaller amounts of steel reinforcing bars. A common nanomaterial employed in cement-based composites is nano-silica. This material accelerates the cement hydration due to the generation of calcium-silicate-hydrate (C–S–H) and dissolution of tricalcium silicates (C_3_S) [50]. In addition, this acceleration of cement hydration is caused by the nano-silica acting as a seed for nucleation of C–S–H [50]. Nano-silica can improve the durability, workability and mechanical properties of cement-based materials [51,52,53,54,55,56,57,58,59]. On other hand, nano-Al_2_O_3_ particles can increase the compressive strength of cement-based materials [41,60,61,62,63]. Al_2_O_3_ nanofibers with a dosage of 0.25% by cement weight may enhance the compressive strength of cement-based materials by up to 30% [50]. Another nanomaterial that can be added to cementitious matrices is nano-Fe_2_O_3_. Optimal values of this nanomaterial improve the compressive strength of concrete specimens [64,65]. The cement added with TiO_2_ nanoparticles can be used to build a photocatalytic concrete with self-cleaning and air-purification characteristics [66]. This concrete type can allow effective photocatalytic decomposition of pollutants, including volatile organic compounds, carbon monoxide, chlorophenols and aldehydes generated from automobiles and industrial emissions [66,67,68]. Also, graphene family nanomaterials can be incorporated into cement composites to enhance their mechanical strength and durability, as well as provide self-sensing abilities [69,70,71,72]. Other novel properties of cement-based materials containing nanomaterials are their low electrical resistivity and self-sensing capabilities [73]. For instance, cement-based composites with CNTs have strain-sensing abilities, which may allow the measurement of their electrical resistance under applied loads [74]. It represents an advantage to obtain strain-sensing concrete structure systems for structural health monitoring [75,76].

This review includes recent studies about the effects on the mechanical strength, durability and workability of cement-based composites due to the incorporation of nanomaterials such as nano-SiO_2_, nano-Fe_2_O_3_, nano-TiO_2_, nano-Al_2_O_3_, CNTs, graphene and graphene oxide. In addition, these studies include nanomaterials that provide self-cleaning and self-sensing abilities to concrete structures. Also, the main challenges of using nanomaterials in cement-based materials are discussed.

## 2. Nanomaterials in Cement-Based Materials

### 2.1. Nano-Silica (Nano-SiO_2_)

**Figure 1:**
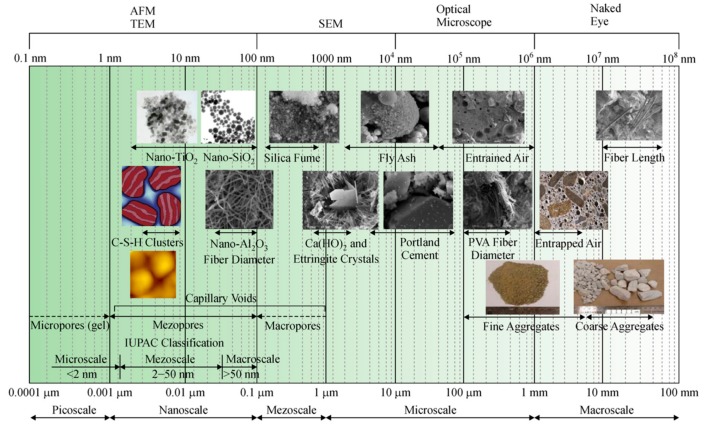
Scale range of several materials used in the concrete fabrication. Reprinted with permission from [77]. Copyright©2017, Higher Education Press and Springer-Verlag, Berlin/Heidelberg.

Nano-silica is a nanomaterial employed for civil engineering applications that can replace micro-silica and silica fume. Nano-silica reacts with lime during the cement hydration process and it generates a C–S–H gel that may improve the mechanical strength and durability of concrete. A good dispersion of nano-silica into cement-based materials can accelerate the hydration process of cement paste, allowing a denser microstructure. On the other hand, an excessive number of nanoparticles can cause agglomeration due to their high surface energy, which will provide a non-uniform dispersion. Figure 1 shows the scale ranges of several materials used in concrete fabrication [77].

Flores-Vivian et al. [77] used Portland cement containing nano-silica to modify the rheological performance and improve the durability and strength. They used a nano-silica content of 0.25% by weight of cement-based materials. Other researchers such as Braz de Abreu et al. [22] reported the use of stabilized nano-silica particles (between 3 and 200 nm in size) in Brazilian-type CP V ARI PLUS Portland cement. They fabricated three types of concrete mixes: a reference concrete, a concrete added with stabilized nano-silica and a concrete including stabilized nano-silica with silica fume. After, they studied the results of concrete compressive strength tests at curing ages of 3, 7 and 28 days. The concrete compressive strength with only stabilized nano-silica increased up to 27%, 20% and 11% at 28 days compared with the reference concrete. On the contrary, the concrete with stabilized nano-silica and silica fume registered even higher compressive strength values (i.e., 28%, 37% and 24% at 28 days) compared to the control concrete. Thus, a mixture of nano-silica and silica fume with Portland cement generated a concrete with higher compressive strength.

Heidari and Tavakoli [78] fabricated a mixture using nano-silica and ceramic powder. They investigated the properties of ceramic power based on the ASTM C 618 standard, using 92% as material in the mixture. In this mixture, the cement is replaced with ceramic powder (phase A). In the second phase (phase B), the ceramic powder percentage is reduced, and the nano-silica is added. They employed the binder content as a constant (320 kg/m^3^) and a water-cement ratio of 0.5. During phase A, mixtures were made with a ceramic powder percentage of 0%, 10%, 15%, 20%, 25%, 30% and 40% of the cement weight using the same proportion of aggregates and water. During phase B, mixtures are made with 0.5% and 1% of nanosilica and different ceramic powder content of 10%, 15%, 20% and 25% of the cement weight. All concrete mixtures are fabricated considering the ASTM C192 standard. The results of the compressive strength tests of the concrete (phase A) were obtained with different curing ages (7, 28, 56 and 91 days). These results show that the concrete strength proportionally decreases with the amount of ceramic powder added to the concrete. The concrete specimen containing 1% of nano-silica and 10% of ceramic powder improved its compressive strength. The impact on the pozzolanic reaction of nano-SiO_2_ is more effective at an early age.

Supit and Shaikh [79] determined the durability properties of high-volume fly ash concrete with addition of nano-silica. They used type I Portland cement and different series of mixtures with a water-cement ratio of 0.40. Compressive strength tests for all concrete mixtures were measured at ages of 3, 7, 28, 56 and 90 days. The incorporation of nano-silica into ordinary concrete increased the compressive strength reaching up to 150% more at early ages. For ages of 28, 56 and 90 days, the compressive strength showed an increment between 45% and 75%. The nano-silica accelerated the hydration process and allowed a cementitious matrix with denser microstructure. The 4% nano-silica- modified concrete decreased its water absorption (between two and three times lower) in comparison to concrete without nano-silica. The resistance of chloride penetration was studied at ages of 28 and 90 days, in which the mixture with 2% nano-silica registered the lowest penetration value. Based on microstructure analysis, nano-silica-modified concrete mixtures presented denser microstructures. Thus, nano-silica modified concretes could be classified as low permeability concretes.

**Figure 2:**
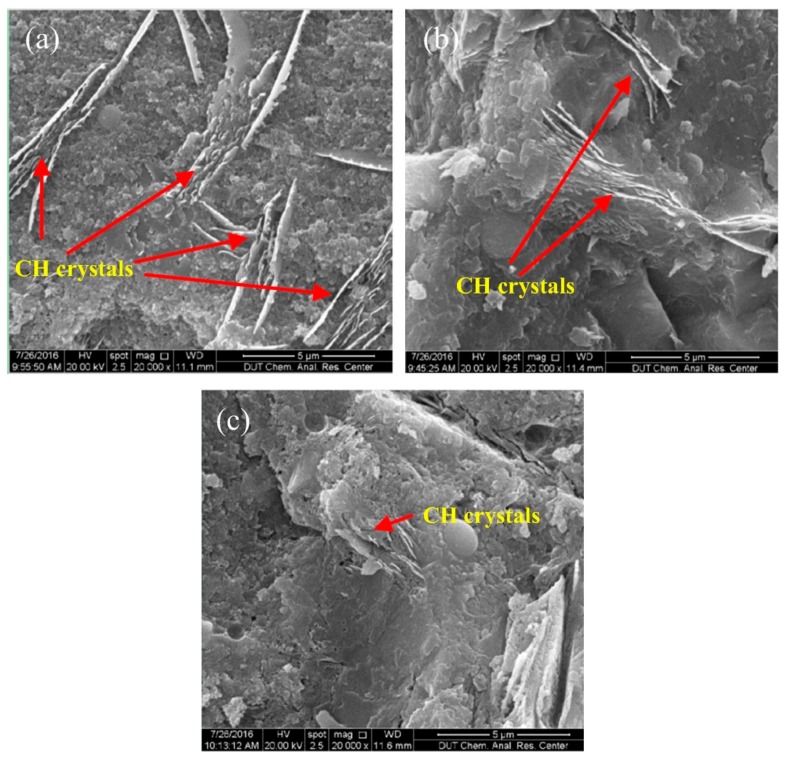
SEM micrographs of CH crystals in concrete with nano-SiO_2_-coated TiO_2_ at curing age of 28 days (20,000×): (**a**) sample T0; (**b**) sample T3; (**c**) sample T5. Reprinted with permission from [80]. Copyright©2017, Elsevier B.V.

**Figure 3:**
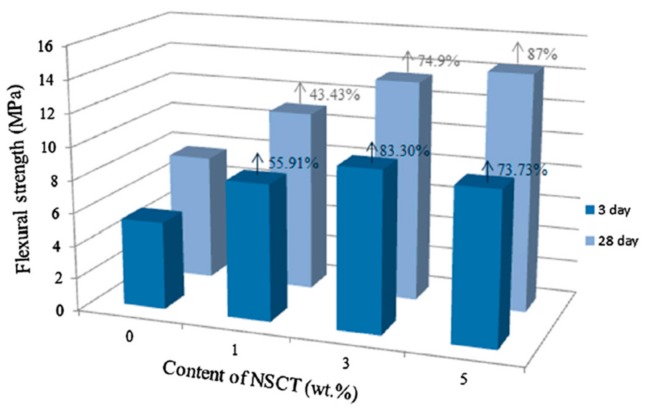
Flexural strength of NSCTRRPC test specimens with different values of NSCT content at curing age of 3 and 28 days. Reprinted with permission from [80]. Copyright©2017, Elsevier B.V.

**Figure 4:**
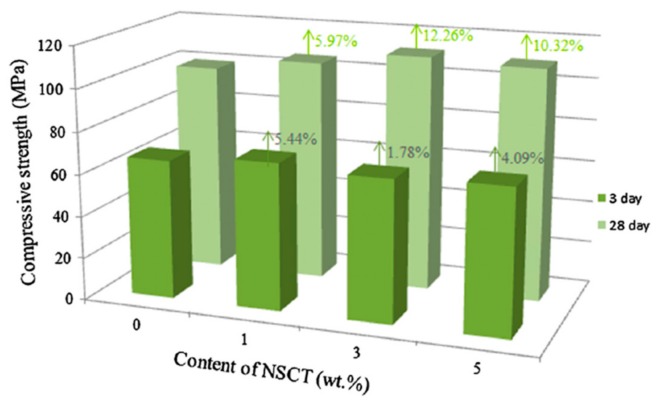
Compressive strength of NSCTRRPC test specimens with different values of NSCT content at curing age of 3 and 28 days. Reprinted with permission from [80]. Copyright©2017, Elsevier B.V.

In order to reinforce reactive powder concrete (RPC), Han et al. [80] added nano-SiO_2_-coated TiO_2_ (NSCT) to RPC. These nanomaterials were studied by scanning electron microscopy (SEM), thermogravimetry (TG) analysis and powder X-ray diffraction (XRD). The acceleration of cement hydration due to the effect of the nucleus played a dominant role in the first days. The CH crystals particles size registered a reduction when the content of NSCT was increased (see Figure 2). The flexural and compressive strength of NSCT reinforced RPC (NSCTRRPC) specimens were investigated at curing ages of 3 and 28 days, considering different contents of NSCT (i.e., 1%, 3% and 5% by cement weight). The NSCTRRPC specimens enhanced their flexural and compressive strength in comparison to RPC specimens without NSCT. Figure 3 and Figure 4 depict the flexural and compressive strength of NSCTRRPC specimens at curing ages of 3 and 28 days. For 3% NSCT dosage at a curing age of 3 days, a maximum flexural strength value (9.77 MPa) of the NSCTRRPC specimen was achieved. It represents an increment of 83.3% compared with the RPC without NSCT. For curing age of 28 days and 5% NSCT content, the flexural strength (14.38 MPa) of the NSCTRRPC specimen was increased up to 87% with respect to RPC without NSCT. Thus, NSCT increases the flexural strength of RCP specimens at both early age (3 days) and later age (28 days). The composites with NSCT registered small increments in their compressive strength at curing age of 3 days. On the contrary, maximum levels of the compressive strength of NSCT modified composites were measured at a curing age of 28 days. Thus, the highest compressive strength (111.75 MPa) of NSCTRRPC specimens is obtained with 3% NSCT dosage. This strength value registered an increase of 12.26% in comparison with RPC without NCST. However, the flexural strength of NSCTRRPC specimens had higher increment levels than that of compressive strength for the same test composites. This is caused by the NSCT that significantly enhances the toughness of the RPC [80].

Li et al. [81] examined the properties of ultra-high-performance concrete, which is obtained with particles of nano-limestone (nano-CaCO_3_) and nano-silica. They used type I Portland cement and fly ash, and silica fume as binding agents. The percentages of nano-silica and nano-limestone by cement weight were of 0.5%, 1.0%, 1.5% and 2.0% and 2.0%, 3.0% and 4.0%, respectively. The mixture workability was reduced with respect the control specimen and it was maintained when the amount of nano-limestone is increased. This is due to the small size of nanoparticles that are found on the surface, leaving less water to contribute towards fluidity. The compressive and tensile strength of concretes including nano-limestone and nano-silica were improved with respect to concretes without any additions. The microstructure with highest values of density and mechanical strength was obtained with content levels of 1% nano-silica and 3% nano-limestone, respectively. The mechanical strength of concrete containing nano-silica and nano-limestone is increased with the reduction of the water-cement ratio.

Sadeghi et al. [82] reported non-destructive compressive strength tests of self-compacting concretes added with steel fibers, polypropylene and nano-silica. They employed the ultrasonic pulse velocity technique in concrete to register mechanical strength of concrete specimens. These concrete specimens (100 × 100 × 100 mm^3^) were fabricated based on II Portland cement at ages of 7, 28 and 90 days. In addition, they used 40 different types of mixtures considering 2%, 4% and 6% of replacement with nano-silica and superplasticizer. In the specimens were measured the wave transmission velocity and compressive strength using the exponential relationship between both parameters. An increment of steel fiber volume above 3% increased the wave pulse transmission velocity in the specimens. The compressive strength and wave pulse transmission velocity increased when the percentage of nano-silica achieved above 4% of cement weight; however, both decreased afterwards.

Najigivi et al. [83] implemented tests using ordinary Portland cement and different nano-silica particles types according to average size. They named each one with the letters N and M, which both particle types reached an amorphous structure with a high pozzolanic reaction. These researchers used a water-cement ratio of 0.40, including nano-silica particles with proportions of 0.5%, 1.0%, 1.5% and 2.0% within the N particles and 2% in the M particles. In all the combinations of these tests, both nano-silica particles types decreased the concrete fluidity. The lime-cure concrete with maximum compressive strength was achieved using 2% nano-silica particles of M type with quicklime solution. This concrete reached the maximum values of compressive strength (40.2 MPa, 53.5 MPa and 57.1 MPa) at curing ages of 7, 28 and 90 days. This increment is due to the calcium hydroxide compounds reacted with nano-silica at a superficial level, generating additional C–S–H gel.

**Figure 5:**
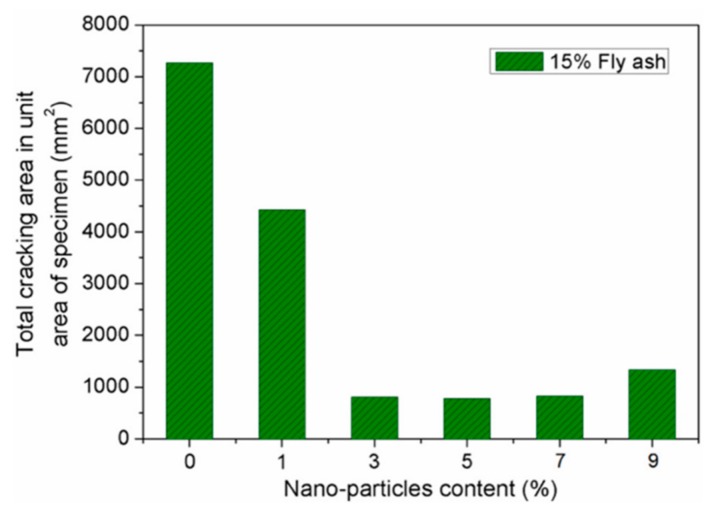
Influence of nano-silica dosage on the total cracking area per area unit of concrete specimens. Reprinted with permission from [84]. Copyright©2019, MDPI AG.

**Figure 6:**
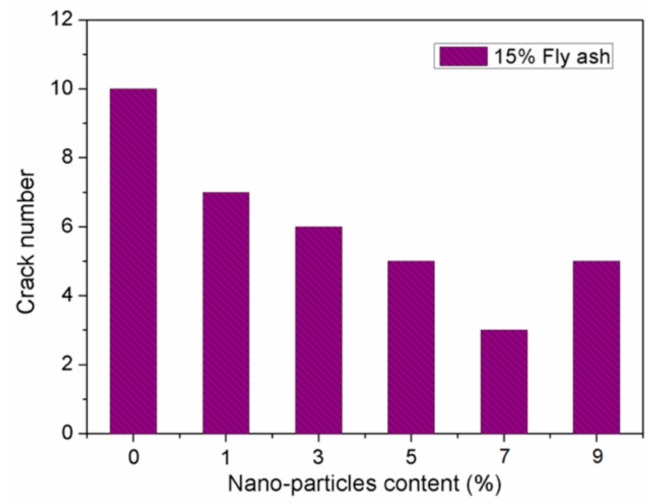
Effect of nano-silica dosage on the cracks number of concrete samples. Reprinted with permission from [84]. Copyright©2019, MDPI AG.

**Figure 7:**
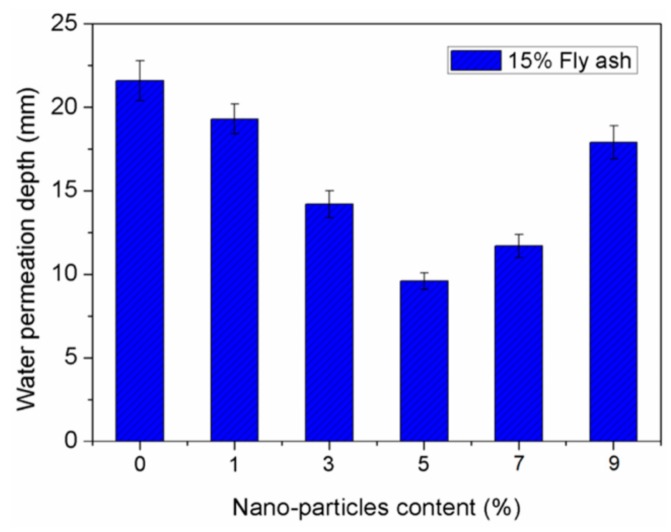
Effect of nano-silica dosage on the water permeation depth of concrete samples. Reprinted with permission from [84]. Copyright©2019, MDPI AG.

Zhang et al. [84] investigated the durability of concrete specimens containing nano-silica and steel fiber. They used five different contents of nano-silica (1%, 3%, 5%, 7% and 9%) and five-volume levels of steel fiber (0.5%, 1%, 1.5%, 2% and 2.5%). The durability tests of concrete specimens included the carbonation and cracking resistance, and permeability and freezing-thawing resistance. The durability tests are examined considering the carbonation depth of the specimens, total cracking area per unit area of the concrete specimen, cracks number, relative dynamic elastic modulus of the samples obtained after of the freezing-thawing cycles, and permeation depth of the water. For instance, a reduction in both the generated cracks number and water permeation depth of the concrete specimens can improve the concrete durability. Figure 5 and Figure 6 show the total cracking area per unit area and cracks number of concrete samples containing 15% fly ash and five different nano-silica dosages. The cracks number in the concrete specimens decreased when the nano-silica dosages increased from 1% to 7%. The minimum number of cracks is achieved with 7% nano-silica dosage, but this number is increased when the nano-silica content is 9%. In addition, the total cracking area significantly decreased for nano-silica contents between 3% and 5%. Although, a nano-silica dosage of 9% caused an increment of 71.8% of the total cracking area compared with 5% nano-silica content. On the contrary, water permeation depth of the concrete specimens is showed in Figure 7. When the nano-silica content increments between 1% and 5%, the water permeation depth of the concrete specimens is significantly reduced. This improvement level decreases for nano-silica dosages of 7% and 9%, respectively. Based on these results, the nano-silica added concrete specimens enhanced their durability when the nano-silica content is within a certain limit. However, a high content of nano-silica could affect the durability of the concrete. 

Tavakoli et al. [85] reported the effect on the compressive strength caused by addition of silica fume and nano-silica in concrete samples at curing age of 7, 28 and 56 days. They used type II Portland cement with different percentages of nano-silica (0.5% and 1%) and silica fume (5% and 10%) by cement weight. For each case, concrete samples containing nano-silica and silica fume increased their compressive strength compared to control specimen without these materials. The concrete samples achieved the highest compressive strength (52.9 MPa) using 10% of silica fume and 1% of nano-silica particles at curing age of 56 days. This strength value is 42.2% higher than that of the concrete sample without nano-silica and silica fume. More investigations about of nano-silica modified cement were reported by Nazerigivi and Najigivi [86]. They studied the influence on the mechanical strength of concrete specimens caused by incorporation of two different nano-silica sizes (15 nm and 80 nm) with percentages of 0.5%, 1.0%, 1.5% and 2.0% by cement weight. They employed ordinary Portland cement and 16 different concrete samples and one control concrete sample for each mechanical test type, as indicated Table 1. A water-to-binder ratio of 0.40 was used into all the concrete samples. With lime solution, these samples are cured at ages of 7, 28 and 90 days. Table 1, Table 2 and Table 3 indicate the measurements of the compressive, split tensile and flexural strength of all the concrete samples. For the three curing ages, the nano-silica added concrete samples improved their compressive strength with respect to that of the control specimen. The compressive strength had a gradual increment when the nano-silica dosage was increased up to 2% of 15 nm plus 1.5% of 80 nm; after, it had a small decrease. The generation of C–S–H gel may be accelerated due to ultra-high specific surface and ultra-fine particle size of nano-silica incorporated in concrete samples [82]. The split tensile and flexural strength of all the nano-silica modified concrete specimens were improved with respect to the control sample. Both split tensile and flexural strength registered gradual increments with the incorporation of nano-silica up to 2% of 15 nm plus 1.5% of 80 nm; after, these mechanical strengths had a small reduction. It could be caused because the total quantity of nano-silica is higher than that to obtain the lime-silica hydration reaction [82].

Mastali and Dalvand [87] reported a theoretical and experimental study of the effects on the mechanical properties of concrete samples due to the presence of 1.0% nano-silica and 7% silica fume, respectively. They realized 270 tests with different designs of self-compacting concrete, in which the impact resistance and mechanical properties of concrete samples were enhanced. The incorporation of nano-silica and silica fume in the cement of silica fume and self-compacting concrete increased 70% its impact resistance for the first crack. Fiber reinforced specimens with water-cement ratio of 0.34 and 0.27 registered the highest average of tensile and flexural strength, respectively. 

Mohammed et al. [88] evaluated the influence on the properties of concrete due to the nano-silica inclusion. This nano-silica incorporation caused a reduction of 13% in the pore amount of the cementitious paste. They studied the relationships that improved the compression strength of the concrete. The workability was modified negatively, which was not affected with the incorporation of superplasticizer to the concrete paste. When the nano-silica inclusion was increased in the experimentation, the permeability and infiltration rate were reduced based on the SEM results.

The incorporation of nano-silica optimal dosage in concrete samples may improve their compressive, tensile and flexural strength. The nano-silica added in cement with other materials such as polypropylene, glass and steel fibers with fixed proportions can increase the mechanical properties of the concrete. Concretes with nano-silica absorbed Ca(OH)_2_ crystals, filling the voids of the C–S–H structure, leading to a denser microstructure.

### 2.2. Nano-Ferric Oxide (Nano-Fe_2_O_3_)

The optimal addition of nano-Fe_2_O_3_ in concrete specimens may improve their compressive strength. In addition, the volume electrical resistance of cement mortars with inclusion of nano-Fe_2_O_3_ can be altered through the applied load, allowing the measure of compressive stress [73]. It can be used for structural heath monitoring of concrete structures without require additional sensors.

**Figure 8:**
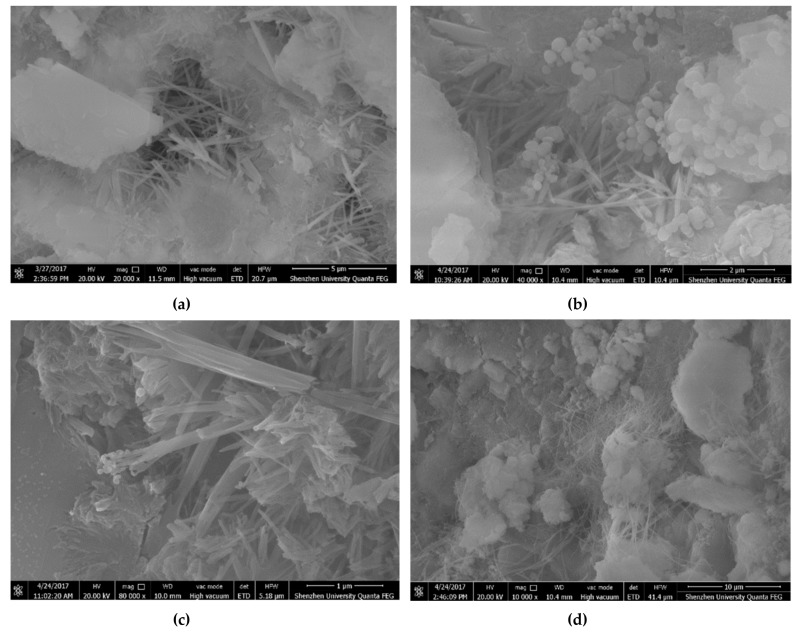
SEM image of concrete specimens with addition of nano-Fe_2_O_3_ (**a**) 0%; (**b**) 3%; (**c**) 5%; (**d**) 10%. Reprinted with permission from [89]. Copyright©2018, Atlantis Press.

Fang et al. [89] measured the mechanical properties of cement samples with different additions of nano-Fe_2_O_3_ (3%, 5% and 10% by cement weight) at ages of 7, 14 and 28 days. Figure 8 shows the SEM image of cement specimens with different additions of nano-Fe_2_O_3_. When the nano-Fe_2_O_3_ content increases then the surface morphology is denser. For all the measurements, the addition of nano-Fe_2_O_3_ in cement mortars increased their compressive strength compared to control mortar. Maximum values of the compressive strength of the cement samples were achieved using 10% of nano-Fe_2_O_3_ content. For this nano-Fe_2_O_3_ dosage at curing ages of 7, 14 and 28 days, the compressive strength of the cement mortar was increased up to 66.81%, 69.76% and 25.20%, respectively.

Rashad [90] presented a review the effects of nano-Fe_2_O_3_, nano-Al_2_O_3_, nano-Fe_3_O_4_ and nanoclay on some properties of cement composites. These properties were the mechanical strength, hydration heat, water absorption, workability, setting time and durability. For instance, the inclusion of nano-Fe_2_O_3_ in the cementitious matrix decreased the water absorption and heat rate values as well as accelerated the peak times. Moreover, the workability of the composite was reduced when the nano-Fe_2_O_3_ content was increased. On the other hand, nano-Fe_2_O_3_ (0.5%–5% in concretes and 0.5%–10% in mortars) added into the cementitious matrix improved the compressive strength. Nazari et al. [91] also studied the workability of concrete including nano-Fe_2_O_3_. For this case, cement was partially substituted with nano-Fe_2_O_3_ (i.e., 0%, 0.5%, 1%, 1.5% and 2% by cement weight) and a water to binder ratio of 0.4 was employed. The workability of concrete is decreased when the nano-Fe_2_O_3_ dosage is increased. In addition, Nazari and Riahi [92] developed two models using genetic programming and artificial neural networks to predict the percentage of water absorption and split tensile strength of concrete samples containing nano-Fe_2_O_3_. 

Khoshakhlagh et al. [93] studied the changes of the concrete properties achieved by adding different percentages (1%–5% by cement weight) of nano-Fe_2_O_3_ and superplasticizer. The flexural, compressive and tensile strength, and the water permeability of the concrete specimens were improved with the incorporation of nano-Fe_2_O_3_ up to 4% by cement weight. The content of nano-Fe_2_O_3_ up to 4wt.% of the concrete specimens increased the coefficient of water absorption. The concrete specimens with nano-Fe_2_O_3_ enhanced their hydration heat, workability and the compressive, flexural and tensile strength.

### 2.3. Nano-Titanium Oxide (Nano-TiO_2_)

The addition of nano-TiO_2_ in concrete specimens can provide self-cleaning properties to the concrete. The concrete containing these nanoparticles can allow a photocatalytic degradation of pollutants (e.g., VOCs, CO, NO_x_, aldehydes and chlorophenols) from industrial and automobile emissions. However, this effect is less efficient with aging due to carbonatation [94,95]. 

**Figure 9:**
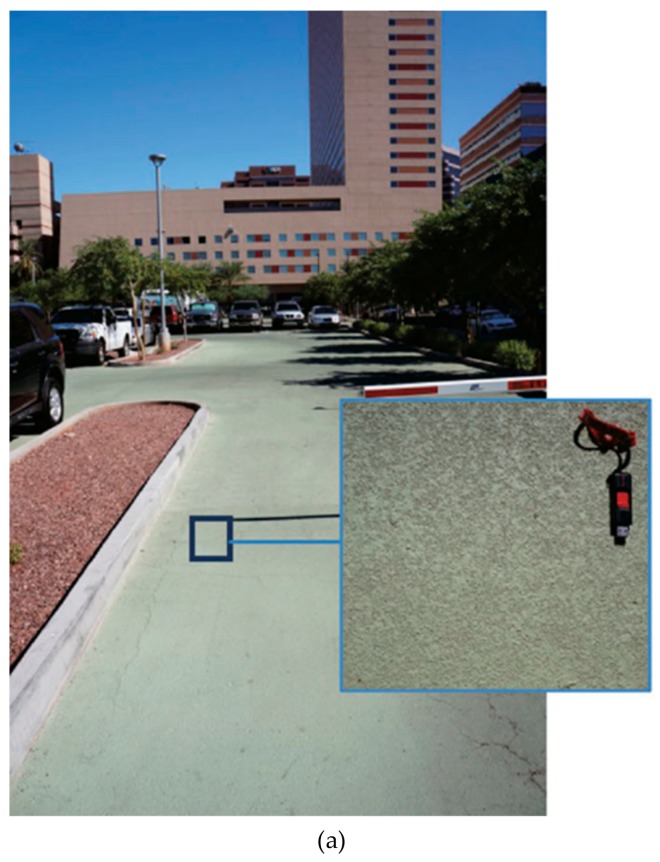

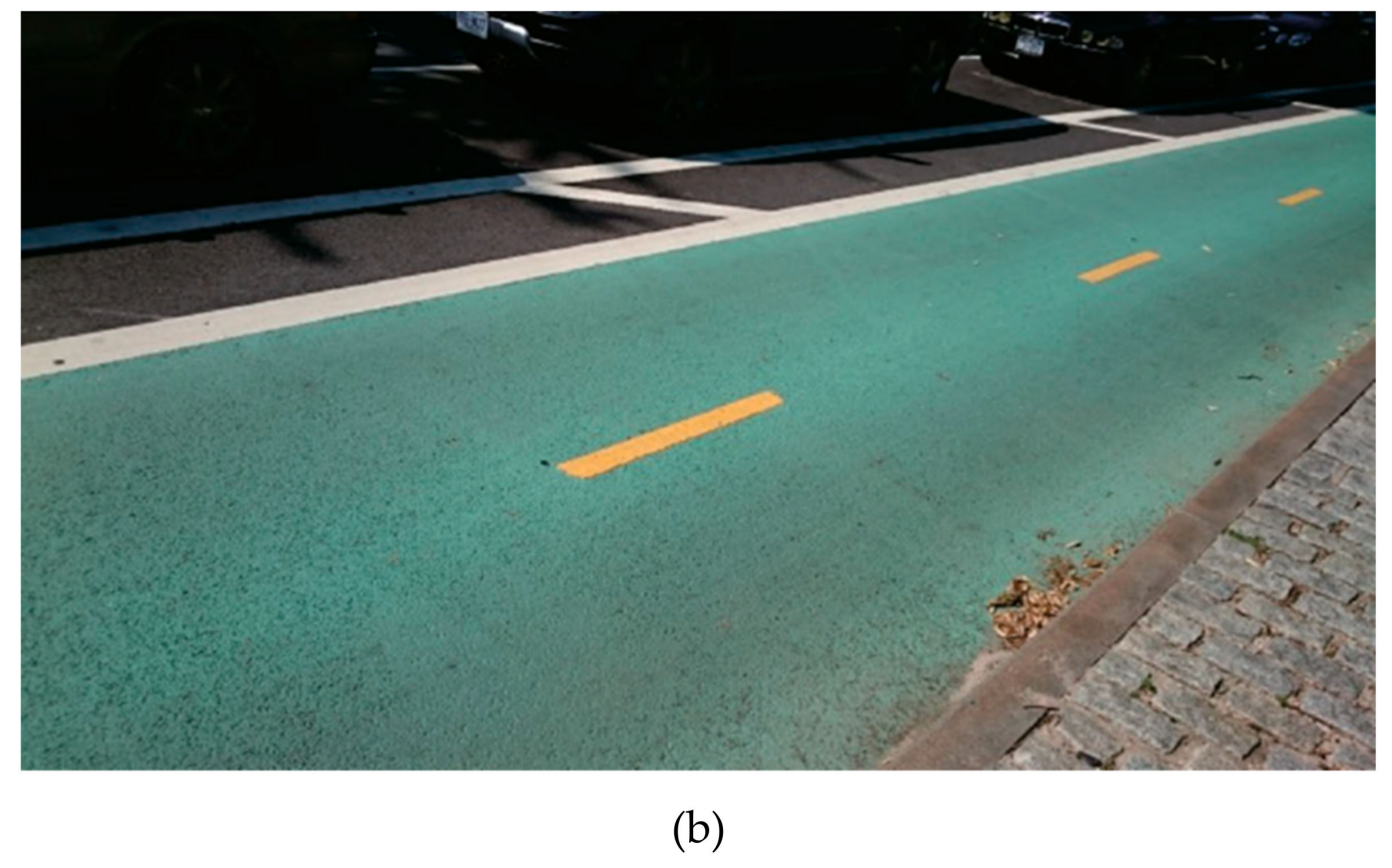
Examples of photocatalytic cement-based coatings that contain TiO_2_ thin film: (**a**) parking lot view (Phoenix, AZ, USA) and (**b**) bike lane (Brooklyn, NY, USA). Reprinted with permission from [23]. Copyright©2016, Higher Education Press and Springer-Verlag Berlin/Heidelberg.

**Figure 10:**
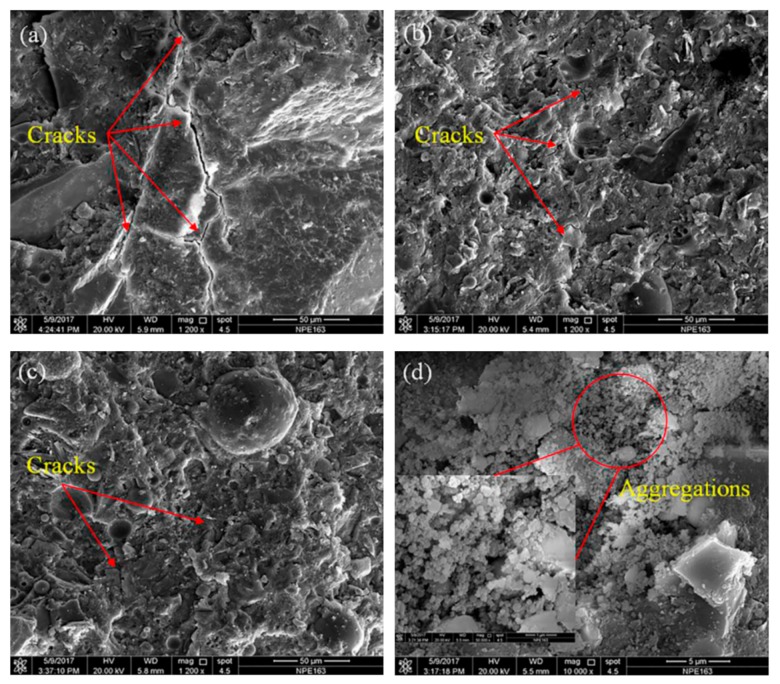
SEM image of the fracture surfaces considering: (**a**) control cementitious composites (1200×); (**b**) nano-TiO_2_ (50 nm size) modified cementitious composites (1200×); (**c**) nano-TiO_2_ (500 nm size) modified cementitious composites (1200×); (**d**) aggregations of nano-TiO_2_ (50 nm size) in cementitious composites (1200×). Reprinted with permission from [96]. Copyright©2019, Elsevier B.V.

**Figure 11:**
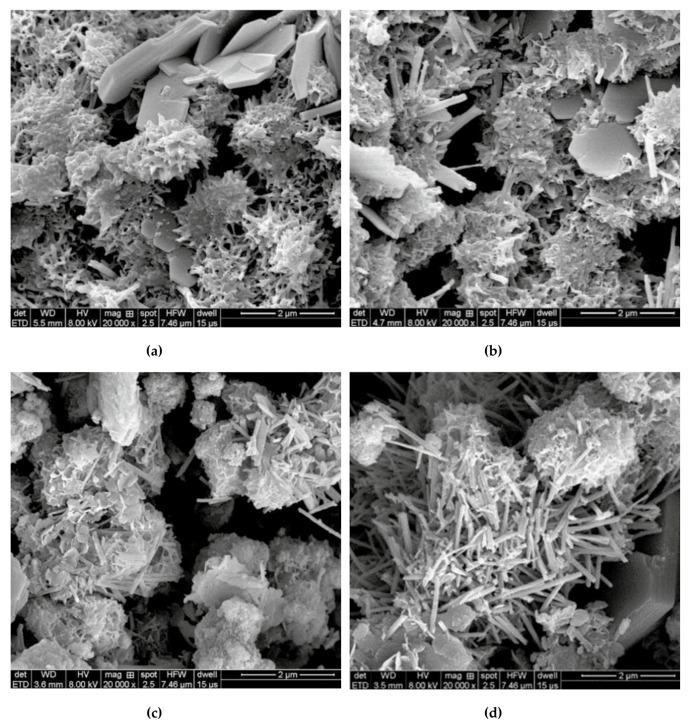
SEM images of cement samples with addition of 2 wt.% nano-TiO_2_ cured under temperatures of (**a**) 0 °C, (**b**) 5 °C, (**c**) 10 °C, and (**d**) 20 °C at 28 days. Reprinted with permission from [97]. Copyright©2018, Hindawi.

**Figure 12:**
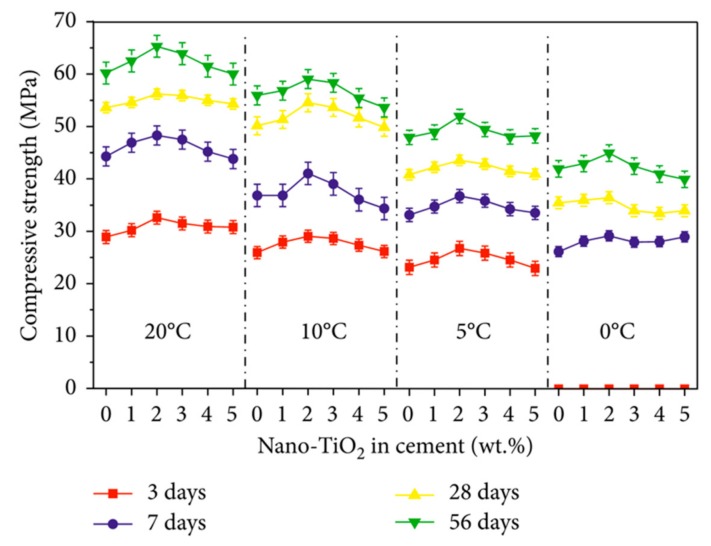
Compressive strength of cement mortar samples incorporating different nano-TiO_2_ dosages. Reprinted with permission from [97]. Copyright©2018, Hindawi.

**Figure 13:**
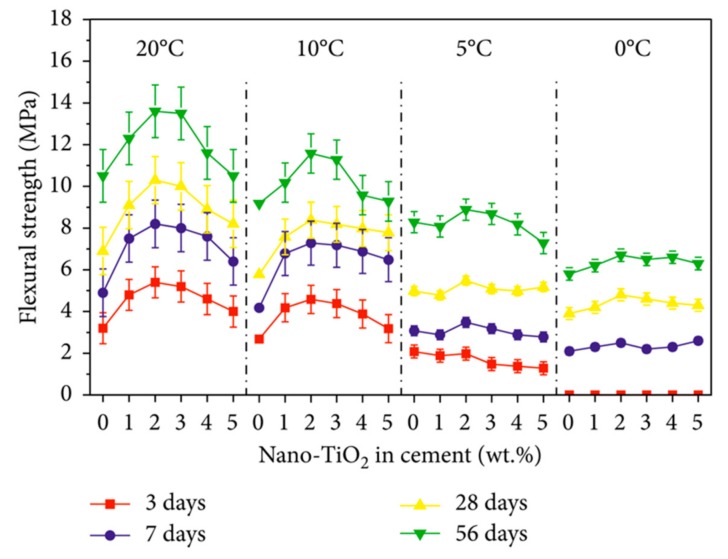
Flexural strength of cement mortar samples containing different nano-TiO_2_ dosages. Reprinted with permission from [97]. Copyright©2018, Hindawi.

Figure 9 depicts two applications of photocatalytic cement-based coatings, which allows a self-cleaning effect in function the decomposition of gases and organic pollutions [23]. This is due to a TiO_2_ thin film on the concrete surface that can provide active oxygen under UV light present in sunlight. Thus, it catalyzes the degradation of organic matters located at the nano-TiO_2_ coated concrete surface [27]. The concrete surface is cleaned with the rainwater, which can prevent the buildup of dirt. Another important characteristic of nano-TiO_2_ is the chemical stability and low price in comparison with other materials. Moreover, nano-TiO_2_ can enhance the resistance to water permeability of cement-based structures [25]. Figure 10 depicts the SEM image of fracture surfaces of cementitious composites considering two sizes of nano-TiO_2_ [96]. Wang et al. [97] investigated the mechanical and physical properties of cement mortar specimens considering different contents of nano-TiO_2_ under curing temperatures of 0, 5, 10 and 20 °C. They used natural river sand, Portland cement (type I ordinary), and TiO_2_ nanoparticles with size of 15 nm. In the experimental tests were used nano-TiO_2_ dosages of 1%, 2%, 3%, 4% and 5% by cement weight, respectively. In the fabrication of the specimens, the nano-TiO_2_ was dispersed in water through ultrasonication. After, cement and sand are mixed during 1 minute. Then, the well-dispersed nano-TiO_2_ was added and mixed during 60 seconds and after water was incorporated. In the following stage, the mortars are collocated into molds and cured using different temperatures. For the specimens were used a water to binder ratio of 0.5. Figure 11 depicts the SEM images of cement pastes including 2 wt.% nano-TiO_2_, at curing age of 28 days under different temperatures. The compressive strength characterization is determined according to ASTM C109 [98] employing a hydraulic testing machine under a controlled load of 1350 N/s. The flexural strength test was evaluated regarding the ASTM C293 [99]. This characterization is determined at curing ages of 3, 7, 28 and 56 days. Figure 11 depicts the results of the hydration degree of the mortar specimens. First, hydration degree of the mortar specimens enhanced through the increment of the nano-TiO_2_ dosage. TiO_2_ nanoparticles can supply an extra space for the precipitation of hydration products. Figure 12 and Figure 13 depict the response of the compressive and flexural strength of the cement mortar samples. Both compressive and flexural strength registered downward trend at low curing temperature. On contrary, flexural and compressive strength of mortar specimens containing nano-TiO_2_ had fast increment with respect to ordinary mortar until that the nano-TiO_2_ content achieved up to 2 wt.%. This increase slowed down for TiO_2_ nanoparticles dosages higher than 2 wt.%. The enhanced strength of mortar samples is caused by TiO_2_ nanoparticles that facilitated the cement hydration and filled the pores in C–S–H gels [97]. These nanoparticles present large surface area to volume ratio, allowing an extra surface area to precipitate hydration products. In addition, TiO_2_ nanoparticles form a bond between them self and C–S–H gel that improves their strength [97].

**Figure 14:**
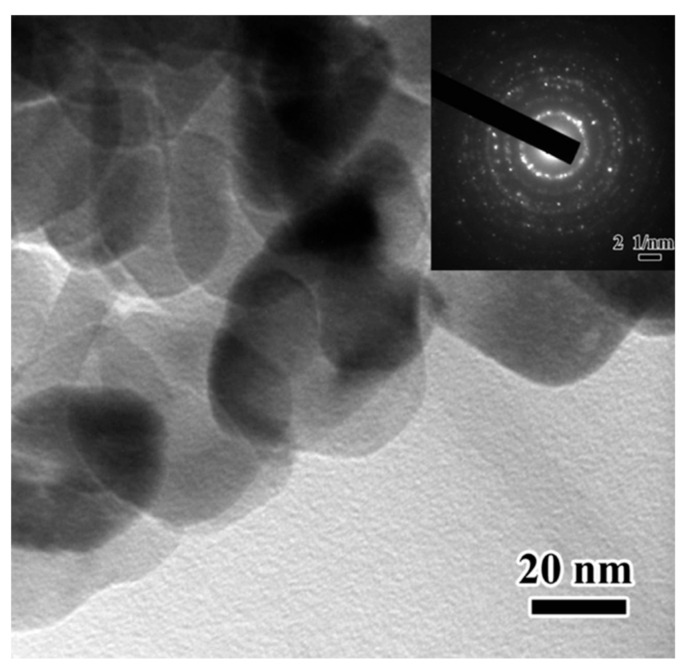
TEM image of the morphology of the TiO_2_ nanoparticles and their selected area electron diffraction (SAED). Reprinted with permission from [100]. Copyright©2013, American Chemical Society.

Feng et al. [100] examined the microstructures of concrete matrices incorporating nano-TiO_2_ as well as the mechanical properties of the cement pastes. Figure 14 is a SEM image of TiO_2_ nanoparticles and their selected area electron diffraction. The incorporation of nano-TiO_2_ (0.1%, 0.5%, 1.0% and 1.5% by cement weight) in cement paste using a water-cement ratio of 0.4 improved the flexural strength (4.52%, 8.00%, 8.26% and 6.71%) at 28 days age.

Jalal et al. [101] studied the characteristics of high resistance self-compacting concrete containing fly ash and nano-TiO_2_. They used Portland cement that was replaced up to 15% weight of waste ash and up to 5% weight of nano-TiO_2_. The addition of nano-TiO_2_ in the concrete improved the consistency and reduced the segregation probability. Considering the water absorption and capillarity, a significant decrease was obtained due to the nano-TiO_2_.

The weight losses in concrete samples were caused by the rapid formation of hydrated products. The self-compacting concrete with nano-TiO_2_ registered a microstructure more refined, which enhanced the resistance to mechanic failures. Other researchers, Yu et al. [102] reported the improvement of concrete microstructure incorporating nano-TiO_2_, which increased its mechanical strength. The TiO_2_ nanoparticles catalyze the decomposition of harmful gases in the air. In addition, the concrete with nano-TiO_2_ achieved a maximum compressive strength that was 7% higher in comparison with the non-added nanoparticle concrete. In addition, Yu et al. [102] investigated the changes of temperatures that can induce cracks and accelerate the hydration reaction. 

Chunping et al. [103] investigated the durability of ultra-high performance concrete due to the incorporation of nano-TiO_2_. This concrete added with 1% nano-TiO_2_ improved its mechanical properties. They investigated the effects on the dry shrinkage, carbonation resistance, freeze-thaw resistance and resistance to chloride ingress. The addition of nano-TiO_2_ in concrete could allow it a self-cleaning and photocatalytic behavior. In addition, the normal concrete containing nano-TiO_2_ could decrease the capillary porosity.

### 2.4. Nano-Alumina (Al_2_O_3_)

**Figure 15:**
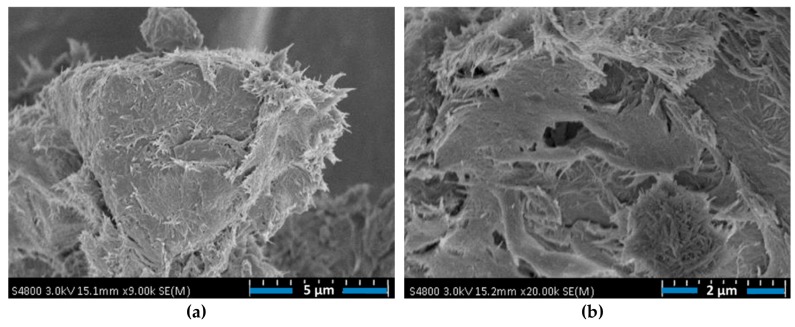
SEM image of the Al_2_O_3_ nanofibers diluted in cement pastes at (**a**) 9000× magnification and (**b**) 20,000× magnification. Reprinted with permission from [50]. Copyright© 2019, Elsevier B.V.

The use of nano-Al_2_O_3_ can accelerate the formation process of C–S–H gel, especially at early-ages, which enhances the strength of composites [104]. For instance, Muzenski et al. [50] fabricated ultra-high strength cement-based materials using Al_2_O_3_ nanofibers with a content of 0.25% by weight of cementitious materials, which improved the compressive strength up to 200 MPa. This represents an increment of 30% in comparison to material strength with only 1% of silica fume. This high compressive strength was achieved with low amount of silica fume. This improved performance is caused by the nanofibers that act as a seed to generate hydration products and contribute the reinforcement for the C–S–H formations, which decrease the number of micro-cracks. In addition, to reach the maximum mechanical performance of the cement-based materials is necessary a suitable dispersion of the Al_2_O_3_ nanofibers. A longer dispersion time could reduce the fibers agglomeration, allowing the enhance of mechanical performance. For instance, the compressive strength at 28 days age achieved higher values for specimens with Al_2_O_3_ nanofibers dispersed for 3 h. Nevertheless, higher quantities of Al_2_O_3_ nanofibers and supplementary cementitious materials did not increase the mechanical behavior of the cement-based materials. Figure 15a,b depicts SEM images of the Al_2_O_3_ nanofibers diluted in cement pastes.

Yang et al. [105] investigated the effect of nano-Al_2_O_3_ on the chloride-binding capacity of cement paste samples. These samples were prepared with nano-Al_2_O_3_ dosages of 0.5%, 1.0%, 3.0% and 5%. The chloride-binding capacity was examined using conventional equilibrium tests, in which the samples were exposed with a NaCl solution at 0.05 mol/L, 0.1 mol/L, 0.3 mol/L, 0.5 mol/L and 1.0 mol/L, respectively. Based on the experimental results, the bound chloride content had an increase of 37.2% at NaCl solution (0.05 mol/L) by adding 5.0% of nano-Al_2_O_3_. Thus, an appropriate adding of nano-Al_2_O_3_ improved the chloride-binding of cement paste samples. 

**Figure 16:**
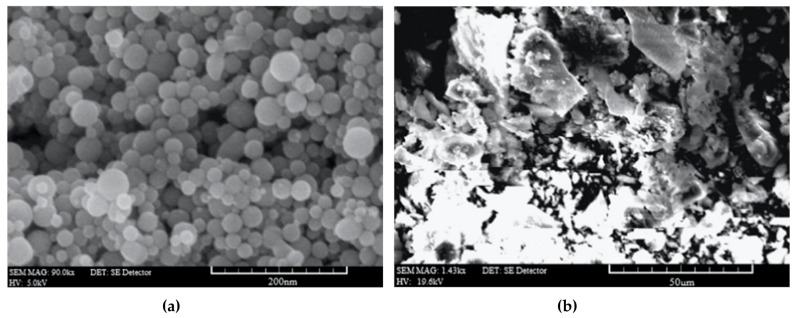
SEM image of the (**a**) nano-Al_2_O_3_ and (**b**) rice husk ash. Reprinted with permission from [106]. Copyright© 2016, Elsevier B.V.

Mohseni at al. [106] studied the effects of nano-alumina and rice husk ash (RHA) in polypropylene fiber (PPF)-reinforced cement mortars. The RHA is an agricultural waste material, which can be recycled to obtain economic and environmental benefits. Figure 16 shows the SEM images of nano-alumina and RHA. The compressive strength of the mortar samples is increased up to 18% and 20% due to the addition of 3% nano-Al_2_O_3_ with 20% RHA at 28 and 90 days. The flexural strength of the mortar samples increased up to 34% and 41% by adding 3% nano-Al_2_O_3_ with 10% RHA. This addition of nano-Al_2_O_3_ generated a denser microstructure in the mortar samples. 

Barbhuiya et al. [107] examined the influence of the incorporation of nano-Al_2_O_3_ on the microstructural properties of the cement paste hydrated at 7 days age. Ordinary Portland cement is substituted with nano-Al_2_O_3_ powder with 2% and 4% by cement weight and the water-cement ratio is fixed to 0.4. In this early-age, they did not note changes at the compressive strength of the cement specimen at early age. 

**Figure 17:**
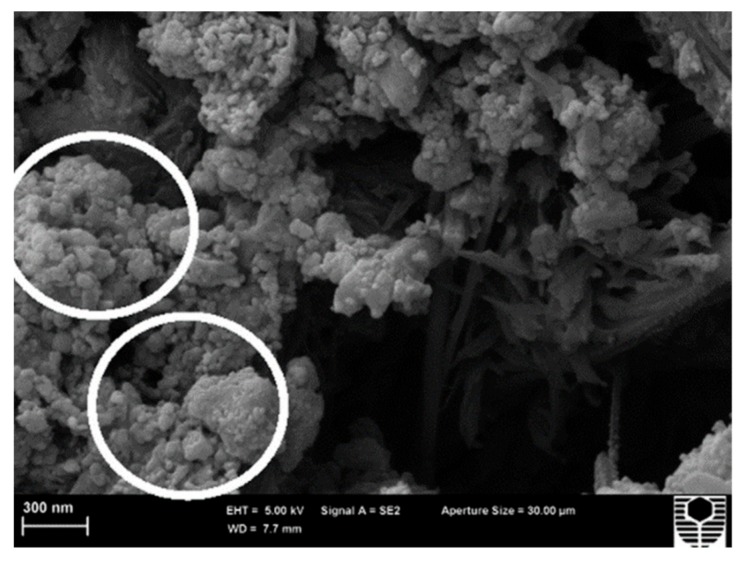
SEM image of 2% nano-Al_2_O_3_ by weight of cement paste hydrated up to 7 days. Reprinted with permission from [107]. Copyright© 2014, Elsevier Ltd.

**Figure 18:**
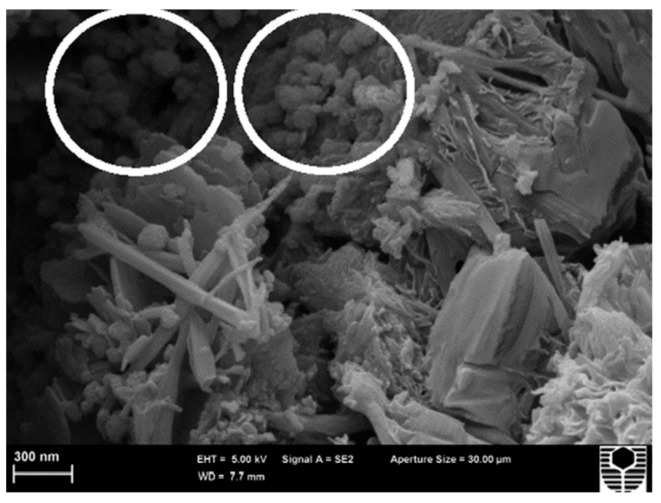
SEM image of 4% nano-Al_2_O_3_ by weight of cement paste hydrated up to 7 days. Reprinted with permission from [107]. Copyright© 2014, Elsevier B.V.

Based on the XRD analysis, Barbhuiya et al. [107] did not find a new crystalline phase developed by adding nano-Al_2_O_3_ within 7 days of curing. They reported the generation of dense microstructure with larger crystal of portlandite within the cement matrix due to the nano-Al_2_O_3_ addition, as shown in Figure 17 and Figure 18. Gowda et al. [108] reported the influence of nano-Al_2_O_3_ in the water absorption and electrical resistivity of cement mortars. They used 1%, 3% and 5% of nano-Al_2_O_3_ by cement weight. The water absorption had a small reduction with the addition of 1% and 3% nano-Al_2_O_3_. However, the water absorption registered a small increment with the addition of 5% nano-Al_2_O_3_. The highest electrical resistivity of the cement mortar is achieved with 5% nano-Al_2_O_3_.

### 2.5. Carbon Nanotubes (CNTs)

Recently, several researchers [109,110,111,112,113,114,115,116,117,118,119,120,121,122,123] have reported the effects of CNTs on the electrical and mechanical properties of concrete samples. For instance, CNTs can decrease the formation and growth of micro-cracks in concrete. The CNTs have important mechanical and electrical properties, including their high strength and high conductivity. For instance, CNTs have high mechanical performance with high aspect ratios (length to diameter ratio) that may generate stronger cement composites [27]. The CNTs cement-based composites have strain-sensing behavior that can measure their electrical parameters under applied loads [124]. This behavior can allow the development of strain-sensing systems of concrete structures for potential applications of damage detection and structural health monitoring [75,125].

**Figure 19:**
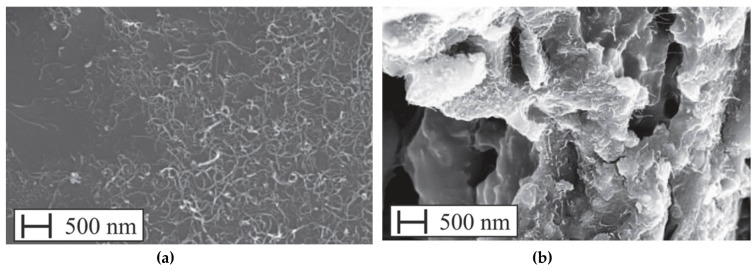
SEM images of the MWCNTs dispersion in (**a**) water suspensions after sonication and (**b**) in a mortar specimen after curing. Reprinted with permission from [124]. Copyright© 2017, Elsevier B.V.

**Figure 20:**
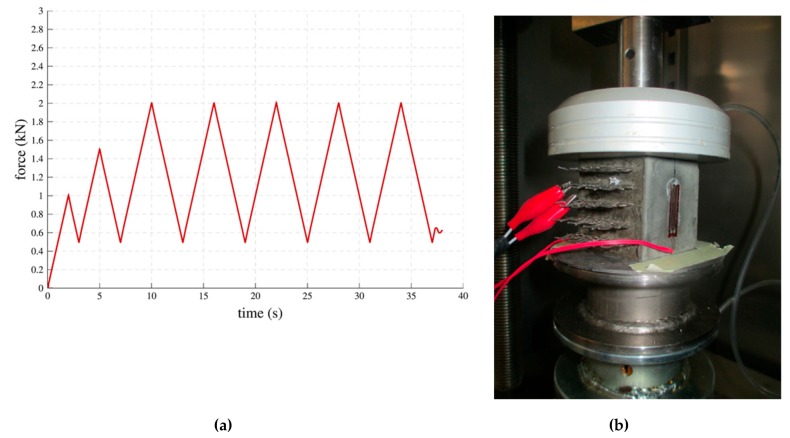
(**a**) Compression load versus time and (**b**) uniaxial testing machine used in the MWCNTs reinforced cement-based specimens. Reprinted with permission from [124]. Copyright© 2017, Elsevier B.V.

**Figure 21:**
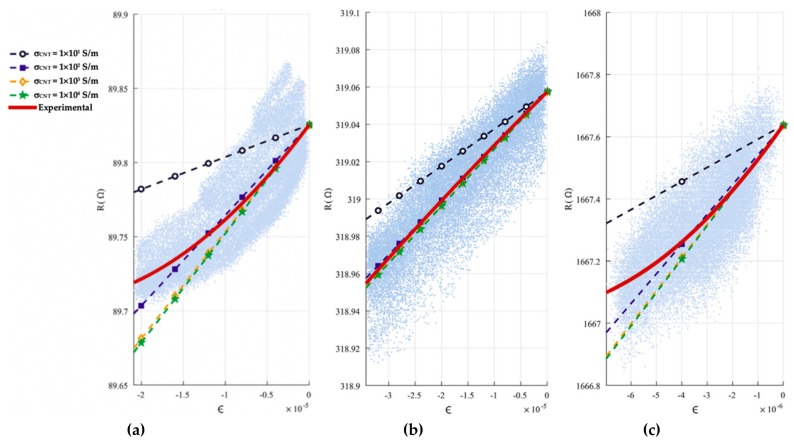
Results of the theoretical and experimental electrical resistance versus applied mechanical strain for (**a**) cement paste, (**b**) mortar and (**c**) concrete samples. Reprinted with permission from [124]. Copyright© 2017, Elsevier B.V.

García-Macías et al. [124] developed a micromechanics model to determine the piezoresistive behavior of cement-based nanocomposites incorporating CNTs and considering the waviness and non-uniform distributions of nanoinclusions. In order to validate the theoretical model, they tested cement-based samples that were doped with multi-walled CNTs (MWCNTS) and exposed to uniaxial compression. These samples were fabricated of concrete, mortar and composite cement paste. Figure 19 depicts the SEM pictures of the MWCNTs dispersion in water solution after sonication and in a cement mortar sample. For the compression loads on the MWCNTs reinforced cement-based composites, they used an equipment of servo-controlled pneumatic universal testing with load capacity of 14 kN, as shown in Figure 20a,b. For the cement paste, mortar and concrete samples are incorporated MWCNTs with electrical conductivity between 10^1^ and 10^4^ S/m. Figure 21a–c illustrates the response of the electrical conductivity of different cement-based composites using the theoretical model and experimental setup. The cement paste, mortar and concrete specimens used filler concentrations of 1%, 0.75% and 0.75% by cement weight. The proposed analytical model may predict the electrical resistance performance of MWCNTs reinforced cement-based materials under compression loads. Ruan et al. [118] reported the influence of different types and dosages of MWCNTs on the mechanical properties of RPC under water or heat curing. They fabricated RPC including four types of MWCNTs with dosages of 0%, 0.25% and 0.50% with water/heat curing, respectively. The mechanical performance of the MWCNTs filled RFC specimens were examined. This mechanical performance considered the flexural strength, fracture energy, compressive/ toughness and flexural strength to compressive strength ratio. The fabrication of the RPC specimens included MWCNTs, water, water reducer, fly ash, quartz sand, cement and silica fume. The cement, silica fume and quartz sand ratio was 1:0.25:1.1. In addition, 20% of cement was substituted by fly ash to enhance the mobility of the mixtures and decrease the cement amount. The four types of MWCNTs used were classified as T1 (functionalized MWCNTs with carboxyl groups), T2 (functionalized MWCNTs with hydroxyl groups), T3 (helical MWCNTs through catalytic cracking) and T4 (nickel-coated MWCNTs).

**Figure 22:**
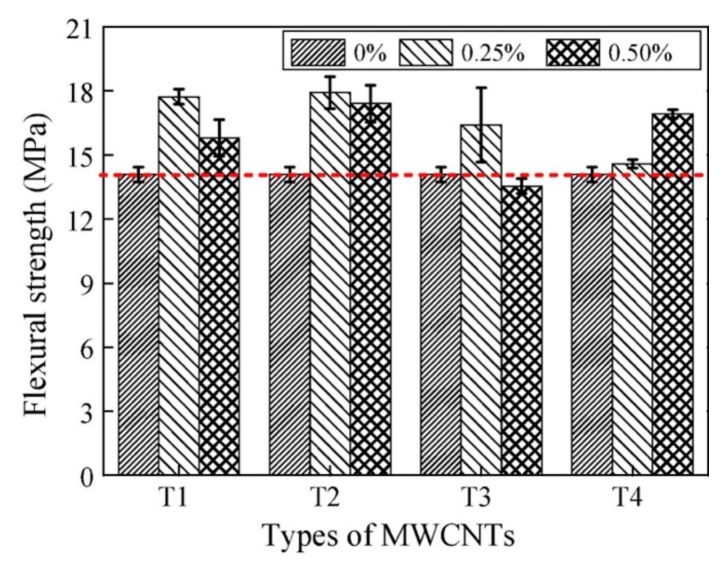
Flexural strengths of the MWCNTs reinforced RPC specimens under water curing. Reprinted with permission from [118]. Copyright© 2018, Elsevier B.V.

**Figure 23:**
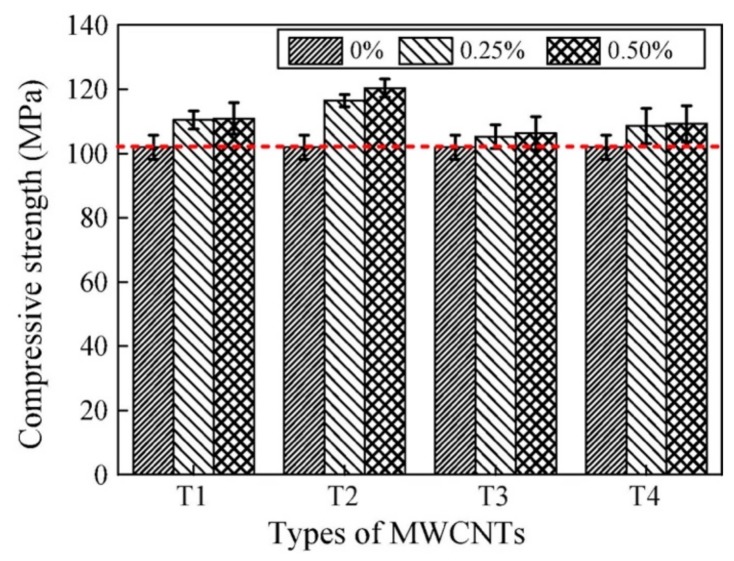
Compressive strengths of the MWCNTs reinforced RPC specimens under water curing. Reprinted with permission from [118]. Copyright© 2018, Elsevier B.V.

**Figure 24:**
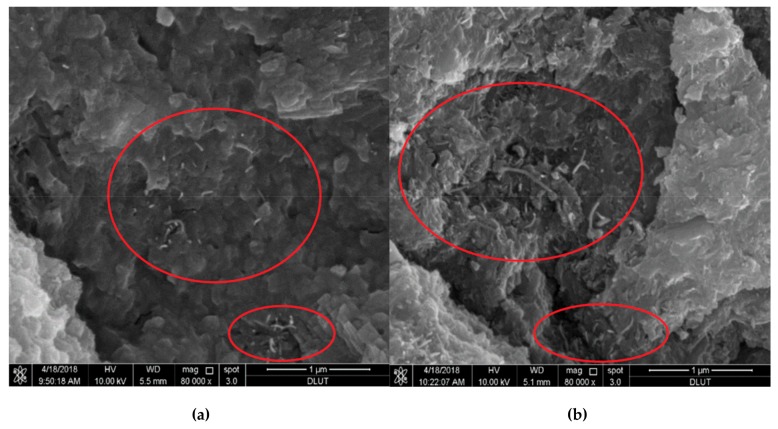
(**a**,**b**) SEM images of the extensive distribution of MWCNTs in RPC. Reprinted with permission from [118]. Copyright© 2018, Elsevier B.V.

Figure 22 illustrates the flexural strengths of the MWCNTs filled RPC under water curing. With exception of the specimen filled with 0.5% MWCNTs dosage of T3, all the others specimens filled with dosages of 0.25% and 0.50% MWCNTs showed enhanced flexural strength. The specimen T2 with 0.25% MWCNTs content had the maximum increase (27.2%) of the flexural strength. However, the specimen with 0.50% MWCNTs content registered a decrease (3.8%) of the flexural strength. For the specimens T1, T2 and T3, the flexural strength had better results for low dosage of MWCNTs than that by high dosage of MWCNTs. In addition, the incorporation of the four types of MWCNTs with dosages of 0.25% and 0.50% improved the compressive strength of the RPC specimens under water curing. The compressive strength increased 18.1% with the incorporation of 0.50% MWCNTs content, compared with the RPC without MWCNTs. The dosage of MWCNTs improved the compressive toughness of all the RPC specimens with water curing, as shown Figure 23. The highest compressive toughness was measured in the specimen T2 with 0.25% MWCNTs content, which represented an increase of 39.2% in comparison to the RPC without MWCNTs. Figure 24a,b depicts SEM images of the wide distribution network of MWCNTs in RPC, which can improve the mechanical properties of RPC.

Lushnikova and Zaoui [120] studied the effect of different types of CNTs incorporated into cement specimens. They used molecular dynamics simulations to determine the influence of CNTs on the mechanical properties of C–S–H such as shear modulus, bulk modulus, elastic constants and Poisson ratio. The results of these simulations registered an improvement of all the studied mechanical properties. Thus, the CNTs are nanomaterials that could enhance the mechanical properties of concrete. Moreover, Sedaghatdoost and Behfarnia [121] examined the influence on the mechanical properties of the Portland cement caused by addition of MWCNTs at the ratios between 0 and 0.15% by weight of cement specimens. These specimens were heated using high temperatures (200–800 °C). The incorporation of 0.1% MWCNT by cement weight improved the compressive, tensile and flexural strength by 35%, 8%, and 11.2%, respectively. In addition, the cement paste was more stable and denser due to the addition of MWCNT. Also, Hawreen and Bogas [122] studied the effects on the long-term creep and shrinkage of concrete due to the incorporation of different types of CNTs. They used concretes with 0.05%–0.5% of unfunctionalized and functionalized CNTs and water to cement ratios of 0.35–0.55. The compressive strength of the concrete with CNTs was increased up to 21%. The addition of CNTs caused a reduction in the early and long-term shrinkage of concrete up to 54% and 15%, respectively. The concrete with addition of CNTs had 17%–18% lower long-term creep in comparison to the concrete without CNTs. Carbon nanotubes are innovative materials for the construction industry that can decrease the formation of nano-cracks. Moreover, the inclusion of CNTs in concrete may increase the compressive and flexural strength of the concrete.

### 2.6. Graphene-Based Nanomaterials

Recent studies [126,127,128,129,130,131,132,133,134,135,136,137,138,139,140,141,142,143,144,145,146,147] have examined the performance of cement-based materials incorporating graphene family nanomaterials (GFN) such as graphene, graphene oxide (GO), reduced graphene oxide (rGO), and graphene nanosheets (GNS). These nanomaterials have extraordinary electrical, mechanical, chemical and thermal properties. Thus, GFN reinforced cement-based materials can improve their structural strength and durability, as well as allow self-cleaning surfaces and self-sensing abilities [148,149,150,151,152,153,154,155,156,157,158].

**Figure 25:**
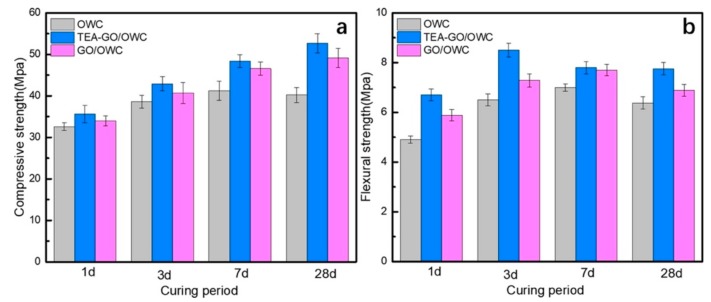
Response of the (**a**) compressive and (**b**) flexural strength of OWC composites containing GO and TEA-GO. Reprinted with permission from [159]. Copyright© 2019, Elsevier B.V.

**Figure 26:**
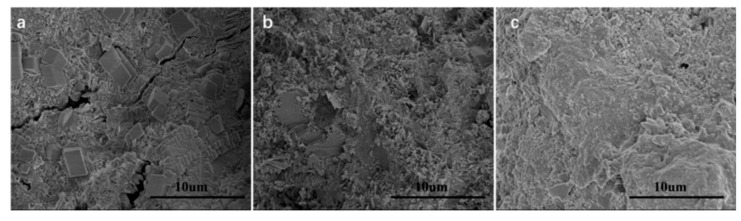
SEM image of the (**a**) blank OWC sample, (**b**) GO reinforced OWC specimen and (**c**) TEA-GO reinforced OWC specimen after compressive strength test at age 28 days. Reprinted with permission from [159]. Copyright© 2019, Elsevier B.V.

**Figure 27:**
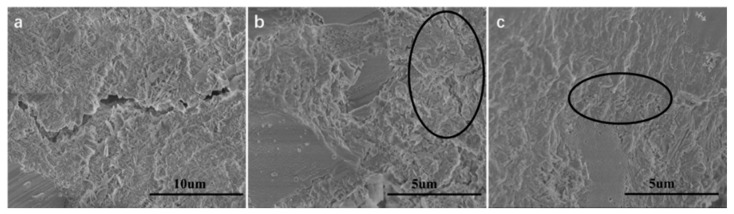
SEM images of the (**a**) plain OWC matrix including a straight-through crack, (**b**) GO/OWC specimen containing fine cracks and (**c**) TEA-GO/OWC specimen. Reprinted with permission from [159]. Copyright© 2019, Elsevier B.V.

Hu et al. [158] fabricated cement composite including nano-silica coated GO, which enhanced its mechanical properties. The compressive strengths of cement composites containing GO and nano-silica coated GO were studied at curing ages of 1, 3, 7 and 28 days. The nano-silica coated GO reinforced cement composites increased their compressive strength up to 120.6%, 124.1%, 126.7% and 133% compared to plain cement with curing ages of 1, 3, 7 and 28 days, respectively. For the GO reinforced cement composites without nano-silica, their compressive strengths improved up to 106.0%, 106.7%, 112.2% and 113.6% with respect to plain cement at curing time of 1 day, 3 days, 7 days and 28 days. The coated nano-silica on GO allowed a finer surface structure and better dispersion, which helped to eliminate the agglomeration of GO in pore solution. The nano-silica coated GO promoted the deposition and growth of C–S–H, refining the cement composite microstructure and improving their macro-mechanical properties. Moreover, Hu et al. [159] functionalized GO via triethanolamine (TEA), which was added into oil well cement (OWC) to enhance its mechanical behavior. The incorporation of TEA-GO and GO was at 0.3 wt.%, keeping a fixed water-to-cement ratio of 0.44. First, they mixed GO/TEA-GO power with water and after added cement within 15 s. In the second stage, the cement was collocated in the cup of Waring blender at 4000 rpm and after was mixing during 15 s at 120,000 rpm. Finally, the cement composites are cured at 60 °C for 1, 3, 7 and 28 days. Both GO and TEA-GO had good dispersion in water; although, TEA-GO with smaller size reported better uniformity. The TEA-GO incorporated to cement allows nucleation sites and acts as seeds to provide the cement hydration. In order to evalue the influence of GO/TEA-GO on the mechanical properties of cement composite, compressive and flexural strength were obtained through mechanical hydraulic pressure testing machine. For the compressive strength tests, the TEA-GO reinforced samples (50.8 × 50.8 × 50.8 mm^3^) were characterized a loading rate of 1.2 KN/s. On the other hand, the flexural strength tests used TEA-GO reinforced samples (160 × 40 × 40 mm^3^) under a loading rate of 0.2 KN/s. Figure 25 illustrates the average compressive and flexural strengths of GO and TEA-GO reinforced cement samples. The TEA-GO reinforced cement samples presented higher increments (9.4%–31%) of the compressive strength than that of GO reinforced cement (4.1%–17.2% ). This is due to that TEA-GO modified cement provides crystals more mature and fewer pores and cracks compared to blank cement and GO reinforced cement samples (see Figure 26). Therefore, the TEA-GO significantly refines the microstructure of cement specimens. On the contrary, the TEA-GO reinforced cement samples achieved the higher increments (8.1%–36.7%) with respect to those of cement incorporating GO (7.8%–20%). The TEA-GO enhanced the mechanical performance of cement due to the increment of hydration degree and limitation to crack propagation. Figure 27 depicts SEM images of the cracks types in three different OWC composites. In the blank cement sample, the cracks penetrate in a straight-through form. For GO reinforced cement sample, the cracks are thinner with random branches. Finally, the cement containing TEA-GO registers fewer cracks with thinner dimensions.

**Figure 28:**
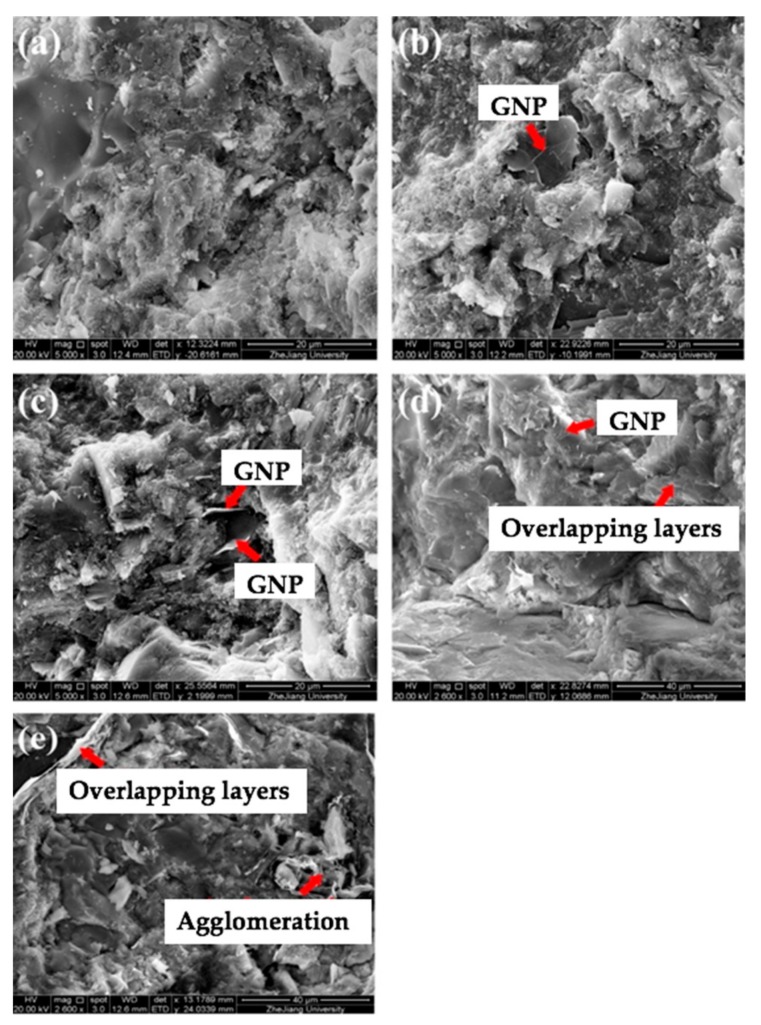
SEM images of the cement mortars added with different GNP content: (**a**) M0 (0%), (**b**) M1 (0.05%), (**c**) M2 (0.1%), (**d**) M3 (0.5) and (**e**) M4 (1%). Reprinted with permission from [160]. Copyright© 2019, Elsevier B.V.

**Figure 29:**
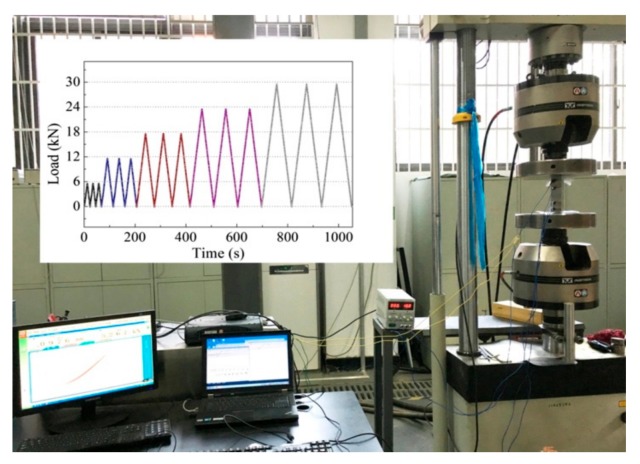
Measurement setup of piezoresistive test of the GNP-added cement mortars under cyclic compressions. Reprinted with permission from [160]. Copyright© 2019, Elsevier B.V.

**Figure 30:**
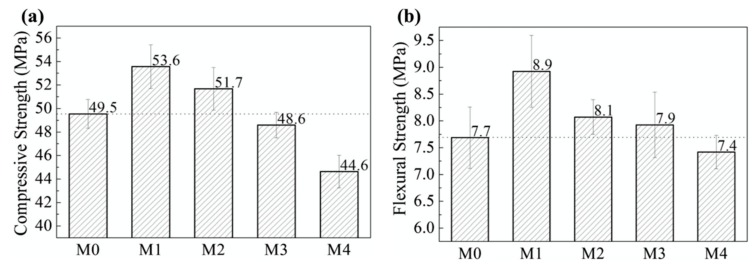
Experimental results of the (**a**) compressive and (**b**) flexural strengths of the GNP reinforced cement mortars. Reprinted with permission from [160]. Copyright© 2019, Elsevier B.V.

Tao et al. [160] investigated the influence of graphene nanoplatelets (GNPs) on the microstructure, pore structure, piezoresistive and mechanical behavior of cement mortar. They quantitatively examined the piezoresistive performance of GNP-reinforced cement mortars exposed to cyclic compressive loads. A PI 42.5 cement is used as binder and natural quartz sands are employed as aggregates. Different dosages (M0 = 0%, M1 = 0.05%, M2 = 0.1%, M3 = 0.5% and M4 = 1%) of GNPs by cement weight were incorporated in cement matrix. The compressive and flexural strength of the GNP-reinforced cement mortars were characterized through a 25 KN high-performance fatigue testing machine. For the compressive and flexural tests were employed loading rates of 144 kN/min and 3 kN/min, respectively. The four-probe method is employed to measure the piezoresistive properties of the cement mortars including GNPs. The cement mortars are dried at 80 °C during 24 h to eliminate the capillary water, which affects the piezoresistive response [161]. Figure 28 illustrates the fracture surfaces of cement mortars containing different GNPs dosages. Later, the cement mortars specimens, regarding the probes and cables, are examined using the mechanical testing machine (see Figure 29). First, the initial resistance of the specimens is determined at stable voltage. Then, the external loads are applied to specimens using constant loading rate of 0.5 kN/s and the piezoresistive properties are obtained through an Instron actuator. Figure 30 depicts the compressive and flexural strengths of the cement mortars. Both strengths firstly increment their values and after decrease when the GNP dosage increases. 

Higher magnitudes of compressive and flexural strengths (53.6 MPa and 8.9 MPa, respectively) are reached in the cement mortar with GNP dosage of 0.25% (M1) by cement weight. These values represent increments of 8.3% and 15.6% compared with the compressive and flexural strengths of the cement mortar without GNP. However, the values of both strengths decrease when the GNP dosages exceed 0.05%. Thus, cement hydrates with homogeneous spatial distribution could be obtained with appropriate values of GNPs dispersed in cement matrix. 

**Figure 31:**
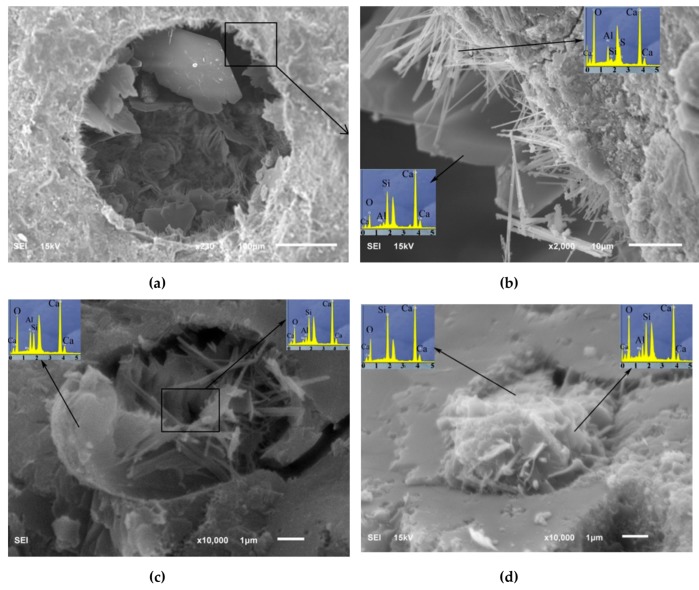
SEM-EDX results of the microstructure of GO composite pores in: (**a**) 0.06% GO composite, (**b**) amplification of (a) showing a surface growth nature of ettringite and flakes structures, (**c**) a small pore of 0.02% GO and (**d**) a small pore of 0.04% GO. Reprinted with permission from [162]. Copyright© 2019, Elsevier B.V.

**Figure 32:**
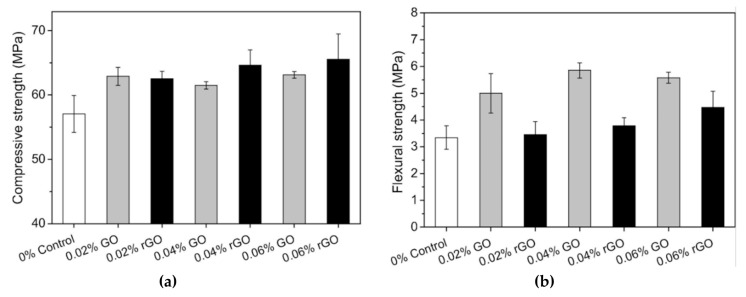
Experimental response of the (**a**) compressive and (**b**) flexural strength of GO and rGO reinforced cement specimens at curing age of 28 days. Reprinted with permission from [162]. Copyright© 2019, Elsevier B.V.

Qureshi and Panesar [162] characterized the influence of GO and rGO on the performance of cement-based composite. They investigated the microstructural properties of GO and rGO using X-ray diffraction (XRD), optical microscope, Fourier-transform infrared spectroscopy (FTIR), SEM, Energy dispersive X-ray (EDS) and Raman spectroscopy techniques. The average C:O ratio of 54.46 and 82.18 in GO and rGO, respectively, were employed in the cement-based materials. To enhance the dispersibility of rGO in water, rGO was processed with superplasticizer. The dosages of both GO and rGO were of 0.02%, 0.04% and 0.06% of cement weight. To reach uniform mixture and efficient dispersion of both GO and rGO in the cement specimens, a water to cement ratio of 0.45 is implemented. In comparison with the control cement sample without GO and rGO, the final setting time and workability of GO reinforced cement specimens gradually decreased using higher GO content up to 0.06% of cement weight. This is caused by the dominant oxygen functional groups and hydrophilic behavior of GO. On the other hand, final setting time and workability of rGO-added cement specimens increased with respect to the control cement sample. It is due to the superplasticizer content and the almost hydrophobic behavior of rGO. The GO composites had greater dosage of C–S–H and Ca(OH)_2_ than the rGO composites at ages of 1, 7 and 28 days. In addition, the GO composites showed micropores filled with crystalline compounds and C–S–H gel. For the rGO composites was found random pore filling nature with ettringite elements. Figure 31 depicts the SEM-EDX results of the microstructure of GO composite pores. Based on the EDX and SEM results of GO and rGO, these nanomaterials present suitable compatibility with cement hydration products, reinforcing the microstructure of the cement composites. Figure 32 depicts the response of the flexural strength and compressive of cement specimens added with GO and rGO at curing age of 28 days. In comparison to control cement sample, cement composites incorporated with 0.02%, 0.04% and 0.06% GO and rGO dosages had an increase of 10.2%, 7.8% and 10.6 %, and 9.6%, 13.3% and 14.9%, respectively. This is due to the high number of functional groups of GO in chemical bonding with cement hydration products and the high mechanical strength of rGO.

Krystet et al. [163] studied the mechanical properties and microstructure of cementitious materials with addition of electrochemically exfoliated graphene (EEG). EEG enhanced the mechanical properties, microstructure and workability of cementitious materials. EEG did not provide aggregate in alkaline environment and the cement mortars incorporating EEG did not decrease its workability and fluidity. The mixture of 0.05 wt.% of graphene with ordinary Portland cement improved the compressive and tensile strength of the cement material up to 79% and 8%, respectively. EEG contributes to hydration reactions of calcium silicates, allowing an intense generation of C–S–H phase and a compact microstructure.

**Figure 33:**
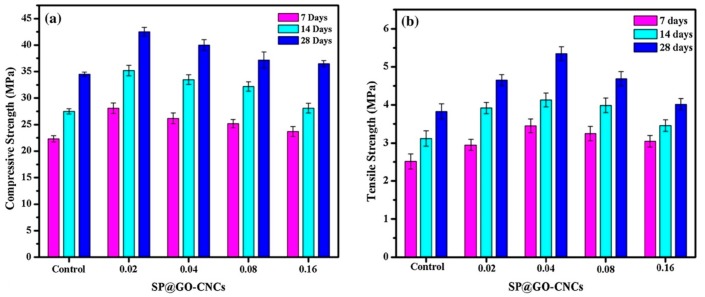
Experimental results of the (**a**) compressive and (**b**) flexural strength of SP@GO modified CNC specimens at curing ages of 7, 14 and 28 days. Reprinted with permission from [164]. Copyright© 2019, Elsevier B.V.

**Figure 34:**
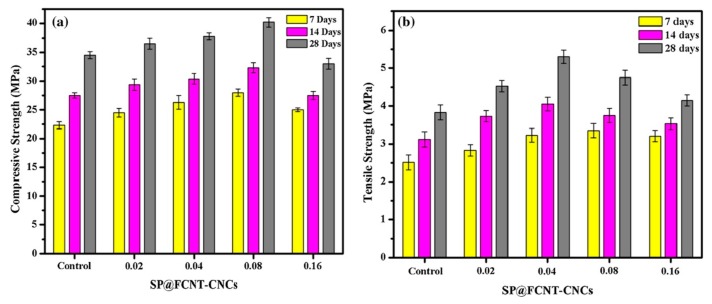
Experimental results of the (**a**) compressive and (**b**) flexural strength of SP@FCNT modified CNC specimens at curing ages of 7, 14 and 28 days. Reprinted with permission from [164]. Copyright© 2019, Elsevier B.V.

**Figure 35:**
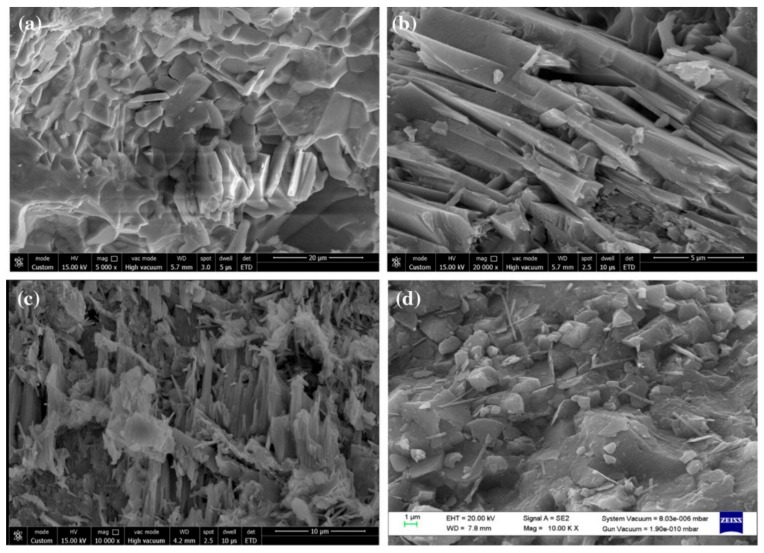
FE-SEM images of CNC specimens incorporating (**a**) 0.02% SP@GO, (**b**) 0.04% SP@GO, (**c**) 0.08% SP@GO and (**d**) 0.16% SP@GO at curing age of 28 days. Reprinted with permission from [164]. Copyright© 2019, Elsevier B.V.

Kaur and Kothiyal [164] compared the effect of polycarboxylate superplasticizer (PCE-SP) added GO and functionalized CNT (SP@GO and SP@FCNT) on the mechanical properties of cement nanocomposites (CNCs). They used two types of SPs to alter the GO and FCNTs structural features, and to enhance the dispersion of these nanomaterials in aqueous solution and cement matrix. The stabilized GO and FCNT allowed to enhance the mechanical strength of the CNC specimens. After, they fabricated three cubes of CNC specimens (70.6 mm × 70.6 mm × 70.6 mm^3^) containing SP@GO and SP@FCNT with different dosages (i.e., 0.02%, 0.04%, 0.08% and 0.16% by cement weight). These specimens were water curing at ages of 7, 14 and 28 days to characterize their mechanical strength. The mechanical tests were done using universal testing machine, applying load at the rate of 3.8 kN/s and 0.5 kN/s, respectively. Figure 33 and Figure 34 show the measurements of average compressive and flexural strength of the SP@GO and SP@FCNT modified CNC specimens. With respect to cement specimen, the maximum values of compressive and tensile strengths of CNC specimens were improved up to 23.2% and 38.5% due to addition of 0.02% and 0.08% SP@GO by cement weight, respectively. On the other hand, addition of 0.08% and 0.04% of SP@FCNT by cement weight enhanced the compressive and tensile strengths of the CNC specimens by 16.5% and 35.8%, respectively. Figure 35 depicts FE-SEM images of CNC specimens containing different SP@GO dosages.

The addition of graphene family nanomaterials in cement composites can enhance their mechanical strength. This will allow the construction of lighter concrete components with extended durability, thus, the consumption of concrete components could be decreased. This will help with the reduction of the gas pollutants resulting from concrete production.

## 3. Challenges

The nanotechnology has allowed the fabrication of nanomaterials that can be incorporated in cement-based materials to generate higher mechanical properties of the concrete structures. The effect of the nanomaterials on the performance of cement-based materials includes the enhance of their compressive, tensile and flexural strength, reduction of the total porosity (i.e., refinement of the microstructure), acceleration of C–S–H gel generation and increment of Young modulus. Furthermore, incorporation of nanomaterials such as nano-TiO_2_ and CNTs can provide self-cleaning and self-sensing properties, respectively, of the products obtained with cement-based materials. To achieve the optimal mechanical properties of the cement-based materials it is very important to mix a suitable dosage of nanomaterial with the cement-based materials. For instance, excessive quantities of nanomaterials added to cement can result in lower compressive, tensile and flexural strength of the cement-based structures. This is caused by nonhomogeneous dispersion of nanomaterials in the cement paste. Thus, the mechanical properties of the nanomaterial reinforced cement-based materials depend of several factors such as the dosage and type of nanomaterial, dispersion method, curing days and curing method. Between these factors, the dispersion method can have a significantly effect on the performance of the nanomaterial reinforced concrete.

**Figure 36:**
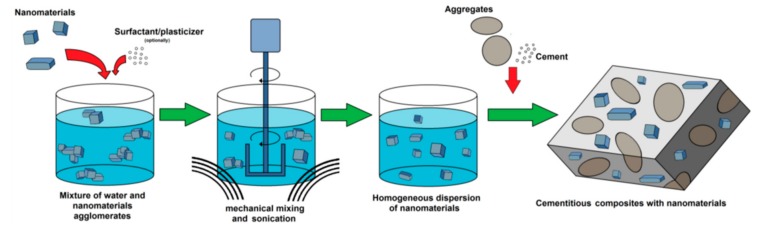
Schematic view of a common nanomaterial dispersion process employed to fabricate a cement-based composite. Reprinted with permission from [167]. Copyright©2018, MDPI AG.

An important challenge for the application of nanomaterials in the construction industry is the development of efficient methods for the dispersion of nanomaterials in cement samples. An alternative solution to incorporate the nanomaterial into cement-based materials consists in the dispersion of the nanomaterial in water before of incorporating it to the dry components of the cement-based materials [165]. For this case, ultrasonic dispersion can be employed as an effective method for the dispersion of the nanomaterials, although, this method requires electrical energy that increases its cost. A bad dispersion of the nanomaterials into cement specimens and the formation of great amount of agglomerates may alter the kinetics of the hydration process, modifying the properties of the cement specimens. For instance, Singh et al. [166] reported that the method used to incorporate nano-silica into cement composites can affect the porosity and mechanical properties of the composites. A bad nano-silica dispersion in cement-based materials may generate voids and weak zones, altering the mechanical properties of the materials. Surfactants such plasticizers and superplasticizers can be used to improve the dispersion of nanomaterials in cement-based materials [51,167,168]. Thus, surface active agents can enhance the homogeneity of dispersion due to the generation of aggregates around nanoparticles [169]. This good dispersion is achieved because of both hydrophobic and hydrophilic groups. The nanomaterials interact with the hydrophobic groups and the hydrophilic groups decrease the water-surface tension, increasing the dispersion of the nanomaterials [167]. Nevertheless, several surfactants (e.g., polymeric matrices) employed for the dispersion of nanoparticles can affect the cement hydration kinetics. Figure 36 depicts a nanomaterial dispersion process employed to obtain a cement-based composite. 

To develop the large scale production of these modified cement-based materials it will be necessary to develop an efficient nanomaterial dispersion method that allows a stable and satisfactory dispersion in cement-based materials [167]. However, the re-agglomeration of the nanoparticles may change their size, which could affect their behavior of nanomaterials in cement-based materials [170]. 

The application nanomaterials in cement-based composites is attractive due to that enhances their mechanical properties only using small dosages of nanomaterials. However, the high cost of these nanomaterials is a limitation to achieve their commercial application in cementitious materials. In the case of graphene and graphene oxide, their fabrication scale is small and relatively expensive. In the future, an important challenge is the reduction of fabrication costs of the nanomaterials. For instance, SiO_2_ nanoparticles may be generated with low cost from hydrothermal solutions generated due the magmatic ore intrusion [77]. Thus, nano-silica and other minerals can be recovered when the steam is condensed in water during the operation of a geothermal power plant [77].

Moreover, another challenge is the optimal dosage determination of the nanomaterial added in the cement paste to obtain the higher mechanical strength and greater durability. To meet this goal, more studies about the effect of different types of nanomaterials on the mechanical properties of cement-based materials are required. These studies must include the effect of the combination of nanomaterials with other cementitious materials. Thus, the optimal quantities of the combination of these materials must be examined to find the best mechanical behavior of the cement-based materials. The main challenge of the use of carbon nanotubes (CNTs)/nanofibers in cement paste is the dispersion due to their strong self-attraction and high hydrophobicity [171,172]. This poor dispersion may cause defects zones in cement/CNTs composites, which constrain the use of CNTs in cementitious matrices [27]. Thus, more investigations must be made with respect to developing methods to improve the dispersion of CNTs in cement-based composites. For example, some researchers [173,174] have studied chemical and surface modification methods for carbon nanotubes to improve their dispersion and bonding between carbon nanotubes and the matrix. Other studies [175,176,177] had examined the mechanical properties of cement samples, which were prepared with different combinations of carbon nanotubes and nano-silica. In addition, future researches must consider prediction models of the relationship between external mechanical deformations and electrical resistivity of cement-based composites incorporating CNTs.

**Figure 37:**
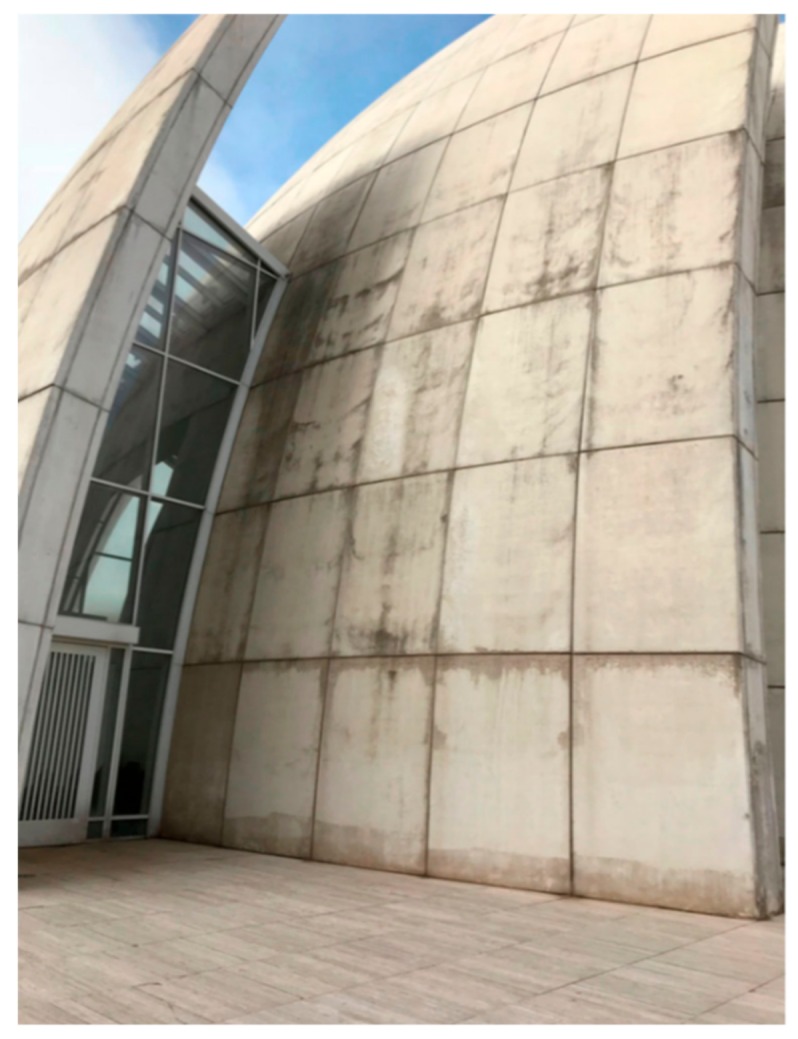
View of the damages on the surface of the self-cleaning concrete shells of Jubilee Church in Rome, which were caused by the local environmental dust. Reprinted with permission from [180]. Copyright©2019, Taylor & Francis Group.

In addition, the future applications of nanomaterials in the construction industry will require one to consider the local environmental conditions (e.g., elements of the local environmental dust). These conditions could damage the performance of the concrete, reducing its durability and increasing the cost of maintenance. For instance, The Jubilee Church in Rome (2003) was one of the first buildings that used self-cleaning and reinforced concrete [178,179,180]. This construction had three iconic shells constructed from 2001 and 2002, which employed precast panels with photocatalytic nano-TiO_2_ particles. Thus, the nano-TiO_2_ particles could absorb energy from light and employ it to achieve a photocatalytic degradation of pollutions. In 2019, Cardellicchio [179,180] reported a study about premature evidence of decay of the three shells that showed failure of their self-cleaning performance. This study considered the material pathologies and their possible damage sources. The surfaces of the shells still contain nano-TiO_2_ particles in the form of anatine, which was detected by this study through chemical analysis. Nonetheless, the self-cleaning properties of the shells with nano-TiO_2_ are only activated when both the sunlight allows the redox of pollutant and the photo-induced hydrophilicity permits the cleaning of the shells [180]. For this case, the hydrophilic characteristic is limited by two main conditions. One condition is linked with common composition of the pozzolanic powder in Rome that cannot be oxidized by titanium dioxide. Another condition is the abrasive effect of the rainwater on the surface of the shells that improves the superficial roughness, increases the bond between powder particles and concrete [180]. The erosive action occurs on the convex surface, which was registered by a colorimetric analysis showing a tendency towards whitish-grey hue caused by the scattering of the sunlight. These two conditions generate a patina which decreases the photocatalytic effect of the surface of the concrete shells (see Figure 37). The efficiency of the nano-TiO_2_ particles incorporated in concrete is affected by the porosity and roughness of the concrete surface. The porosity of the concrete surface may allow the water retention and its roughness may help the adhesion of powder on the surface. The chemical and abrasive characteristics of pozzolanic powder may decrease the efficiency of the self-cleaning of the nano-TiO_2_ added concrete. Therefore, future buildings that use concrete with self-cleaning properties may be affected by powder of volcanic origins or precipitations incorporating desert dust [180].

More investigations about new nanomaterials to improve the mechanical properties of cement-based composites are required. In addition, to achieve the commercial application of these nanomaterials reinforced cement-based composites is necessary to know the effect of the nanomaterials on the mechanical properties of these composites. Therefore, future researches must include the development of novel theoretical models that can predict the mechanical properties of the cement-based materials as function of dosage level of the nanomaterials. Also, more researches about the impact of the nanomaterials in the public health and environment must be developed. There are few studies about the effects of the nanomaterials used in cement-based materials on the public health and environment. For instance, Lee et al. [181] reported some effects of nanoparticles used in the construction industry on environment health and safety. Lam et al. [182] presented a review about the carbon nanotube toxicity, which could produce pulmonary inflammation and cardiac toxicity. Moreover, nano-TiO_2_ could generate inflammation in mammalian cells [183]. Thus, many researches must be done to reduce the negative impacts of the nanomaterials on the health and environment. In addition, more studies about the bioavailability and environment mobility of nanomaterials required to be done [182].

## 4. Conclusions

The incorporation of nanomaterials in concrete can improve their compressive, tensile and flexural strength. Recent investigations had considered nanomaterials such as nano-silica, nano-titania, nano-ferric oxide, nano-alumina, CNT, graphene and GO. The addition of these nanomaterials in concrete can achieved denser microstructures, decreasing the water absorption. The workability of the concrete could be improved by adding these nanomaterials. The nano-TiO_2_ modified concrete can provide it self-cleaning properties and other benefits to help the environment clean. In addition, nano-TiO_2_ added in concrete can allow the photocatalytic degradation of pollutants (e.g.; NO_x_, VOCs, CO, chlorophenols, and aldehydes) from automobile and industrial emissions. The CNTs reinforced cement-based composites can have self-sensing abilities for applications of structural health monitoring or damage detection. In addition, graphene and GO added in cement-based materials can increase their mechanical strength and durability, as well as develop self-cleaning surfaces and self-sensing abilities.

In the construction industry, the fabrication of cement-based composites can generate high levels of CO_2_ gas. To address this problem, one solution is the addition of nanomaterials to cement-based composites, which can provide structural components with high mechanical strength and great durability. Thus, the maintenance requirements and replacement frequency of the cement-based structural components can be decreased. These advantages can allow the reduction of the percentage of cement used in the construction industry. This in turn will decrease the CO_2_ emissions caused by the cement fabrication process. 

The application of nanotechnology in cement-based materials is still in a research stage. The results of experimental tests of nanomaterials-reinforced cement specimens have demonstrated that they can enhance the mechanical strength and durability of the resultng concretes. Moreover, these nanomaterials can allow a novel generation of smart cement-based composites with strain-sensing abilities for damage inspection and structural health monitoring.

## Figures and Tables

**Table 1:** Compressive strength of concrete specimens containing several nano-silica contents. Reprinted with permission from [86]. Copyright©2019, Elsevier B.V.

Sample	Nano-Silica		Compressive Strength (MPa)			Improvement of Compressive Strength (%)		
	15 nm Nanoparticle	80 nm Nanoparticle	7 Days	28 Days	90 Days	7 Days	28 Days	90 Days
C0 (control)	0	0	26.3	34.8	40.3	0	0	0
N1	0.5	0.5	28.2	38.4	44.9	7.2	10.3	11.4
N2	0.5	1	30.3	41.3	49.2	15.2	18.7	22.1
N3	0.5	1.5	32.9	44.2	53.0	25.1	27.0	31.5
N4	0.5	2	35.2	46.8	57.6	33.8	34.5	42.9
N5	1	0.5	31	42.5	50.7	17.9	22.1	25.8
N6	1	1	33.4	46.2	54.1	27.0	32.8	34.2
N7	1	1.5	36.3	47.5	58.2	38.0	36.5	44.4
N8	1	2	40.7	50	63.3	54.8	43.7	57.1
N9	1.5	0.5	35.2	47	56.1	33.8	35.1	39.2
N10	1.5	1	37.2	49.1	59.8	41.4	41.1	48.4
N11	1.5	1.5	41.3	52.2	64.7	57.0	50.0	60.5
N12	1.5	2	46.4	58.7	69.3	76.4	68.7	72.0
N13	2	0.5	39	50.3	63.4	48.3	44.5	57.3
N14	2	1	41.9	54	67.2	59.3	55.2	66.7
N15	2	1.5	52.1	63.7	78.1	98.1	83.0	93.8
N16	2	2	50.3	61	75.2	91.3	75.3	86.6

**Table 2:** Split tensile strength of concrete specimens containing several nano-silica contents. Reprinted with permission from [86]. Copyright©2019, Elsevier B.V.

Sample	Nano-Silica		Split Tensile Strength (MPa)			Improvement of Split Tensile Strength (%)		
	15 nm Nanoparticle	80 nm Nanoparticle	7 Days	28 Days	90 Days	7 Days	28 Days	90 Days
C0 (control)	0	0	1.3	1.5	1.9	0	0	0
N1	0.5	0.5	1.8	2	2.7	38.5	33.3	42.1
N2	0.5	1	2.1	2.4	3	61.5	60.0	57.9
N3	0.5	1.5	2.5	2.9	3.5	92.3	93.3	84.2
N4	0.5	2	3	3.3	3.9	130.8	120.0	105.3
N5	1	0.5	2.2	2.8	3.2	69.2	86.7	68.4
N6	1	1	2.7	3.1	3.8	107.7	106.7	100.0
N7	1	1.5	3.1	3.7	4.2	138.5	146.7	121.1
N8	1	2	3.8	4.2	4.8	192.3	180.0	152.6
N9	1.5	0.5	2.9	3.3	4.1	123.1	120.0	115.8
N10	1.5	1	3.3	3.8	4.6	153.8	153.3	142.1
N11	1.5	1.5	4.1	4.5	5.2	215.4	200.0	173.7
N12	1.5	2	4.4	4.8	5.7	238.5	220.0	200.0
N13	2	0.5	3.6	4	4.9	176.9	166.7	157.9
N14	2	1	4.5	4.8	5.5	246.2	220.0	189.5
N15	2	1.5	4.9	4.3	5.9	276.9	186.7	210.5
N16	2	2	4.3	4.8	5.1	230.8	220.0	168.4

**Table 3:** Flexural strength of concrete specimens containing several nano-silica contents. Reprinted with permission from [86]. Copyright©2019, Elsevier B.V.

Sample	Nano-Silica		Flexural Strength (MPa)			Improvement of Flexural Strength (%)		
	15 nm Nanoparticle	80 nm Nanoparticle	7 Days	28 Days	90 Days	7 Days	28 Days	90 Days
C0 (control)	0	0	4	4.2	4.5	0	0	0
N1	0.5	0.5	4.4	4.5	5	10.0	7.1	11.1
N2	0.5	1	4.6	4.9	5.3	15.0	16.7	17.8
N3	0.5	1.5	5.1	5.2	5.7	27.5	23.8	26.7
N4	0.5	2	5.4	5.6	6	35.0	33.3	33.3
N5	1	0.5	4.6	5.1	5.4	15.0	21.4	20.0
N6	1	1	5.3	5.5	5.9	32.5	31.0	31.1
N7	1	1.5	5.5	5.8	6.3	37.5	38.1	40.0
N8	1	2	5.9	6.2	6.7	47.5	47.6	48.9
N9	1.5	0.5	5.4	5.7	6.1	35.0	35.7	35.6
N10	1.5	1	5.6	6	6.6	40.0	42.9	46.7
N11	1.5	1.5	6.2	6.5	7	55.0	54.8	55.6
N12	1.5	2	6.5	6.8	7.3	62.5	61.9	62.2
N13	2	0.5	5.8	6.2	6.8	45.0	47.6	51.1
N14	2	1	6.5	6.7	7.2	62.5	59.5	60.0
N15	2	1.5	7	7.3	7.8	75.0	73.8	73.3
N16	2	2	6.8	7	7.2	70.0	66.7	60.0
